# Vitamin E beyond Its Antioxidant Label

**DOI:** 10.3390/antiox10050634

**Published:** 2021-04-21

**Authors:** Anca Ungurianu, Anca Zanfirescu, Georgiana Nițulescu, Denisa Margină

**Affiliations:** 1 Department of Biochemistry, Faculty of Pharmacy, “Carol Davila” University of Medicine and Pharmacy, Traian Vuia 6, 020956 Bucharest, Romania; anca.ungurianu@umfcd.ro; 2 Department of Pharmacology and Clinical Pharmacy, Faculty of Pharmacy, “Carol Davila” University of Medicine and Pharmacy, Traian Vuia 6, 020956 Bucharest, Romania; anca.zanfirescu@umfcd.ro; 3 Department Pharmaceutical Technology, Faculty of Pharmacy, “Carol Davila” University of Medicine and Pharmacy, Traian Vuia 6, 020956 Bucharest, Romania; georgiana.nitulescu@umfcd.ro

**Keywords:** tocopherols, tocotrienols, inflammation, cancer

## Abstract

Vitamin E, comprising tocopherols and tocotrienols, is mainly known as an antioxidant. The aim of this review is to summarize the molecular mechanisms and signaling pathways linked to inflammation and malignancy modulated by its vitamers. Preclinical reports highlighted a myriad of cellular effects like modulating the synthesis of pro-inflammatory molecules and oxidative stress response, inhibiting the NF-κB pathway, regulating cell cycle, and apoptosis. Furthermore, animal-based models have shown that these molecules affect the activity of various enzymes and signaling pathways, such as MAPK, PI3K/Akt/mTOR, JAK/STAT, and NF-κB, acting as the underlying mechanisms of their reported anti-inflammatory, neuroprotective, and anti-cancer effects. In clinical settings, not all of these were proven, with reports varying considerably. Nonetheless, vitamin E was shown to improve redox and inflammatory status in healthy, diabetic, and metabolic syndrome subjects. The anti-cancer effects were inconsistent, with both pro- and anti-malignant being reported. Regarding its neuroprotective properties, several studies have shown protective effects suggesting vitamin E as a potential prevention and therapeutic (as adjuvant) tool. However, source and dosage greatly influence the observed effects, with bioavailability seemingly a key factor in obtaining the preferred outcome. We conclude that this group of molecules presents exciting potential for the prevention and treatment of diseases with an inflammatory, redox, or malignant component.

## 1. Introduction

Lipids greatly vary in structure and function, and some, such as liposoluble vitamins (A, D, E, and K) and polyunsaturated fatty acids (PUFAs), constitute essential nutrients because they cannot be synthesized in the human body [1,2]. They and their metabolites can exert direct cellular effects, participate in various cell processes, or yield numerous regulatory functions like signal transduction modulation or gene expression [2,3]. 

Vitamin E, comprising eight vitamers (four tocopherols (TFs) and four tocotrienols (TTs)), is the most abundant liposoluble antioxidant compound in the human body, and its modulatory effects regarding signal transduction, cellular pathways (e.g., NF-κB signaling), and gene expression (e.g., pro-inflammatory cytokines) have recently gained notoriety [3,4,5].

Though several in vitro and in vivo preclinical studies have reported numerous cellular pathways modulated and beneficial effects exerted by vitamin E, human clinical studies have shown sometimes-conflicting results, skewing towards beneficial and protective action of these molecules; notable differences have been found among them, with some of the 8 vitamers proving to be more efficient than others [5].

The inadequate intake of vitamin E is associated with a higher risk of the development of several low-grade inflammation-associated diseases [5]. Low-grade inflammation represents a prolonged inflammatory state characterized by a modest increase of pro-inflammatory molecules (e.g., C reactive protein (CRP)) without the well-known signs of inflammation, with an initial purpose of restoring tissue homeostasis. However, its persistence leads to an alteration or loss of tissue function and is linked to the development of numerous cardio-metabolic (e.g., metabolic syndrome, cardiovascular diseases, type 2 diabetes mellitus, and non-alcoholic fatty liver disease) and neurodegenerative diseases [5,6,7,8,9]. An increased vitamin E intake/dietary supplementation has been linked to beneficial effects regarding the progression and management of these diseases [4,10,11]. Earlier reports focused on the effects of α-TF, as it is the best-represented in plasma [2], but recent reports have shown important regulatory effects of the other vitamers such as δ-TF and γ-TT [3,12]. 

The aim of this review is to present an overview and an update of the molecular mechanisms and signaling pathways involved in inflammation and inflammation-related alterations modulated by vitamin E (TFs and TTs) and/or their main metabolites observed in vitro and in vivo; it also presents preclinical reports and findings in human studies regarding their influence on cardio-metabolic health and anti-neoplastic effects, as well as touching on key bioavailability and metabolism aspects.

## 2. Methods

A survey of literature was performed using PUBMED in order to find the most relevant articles reporting preclinical, in vitro and in vivo, and clinical effects of vitamin E vitamers. Articles were limited to those published in the English language, focusing on most recent works between 2010 and 2021 (64% of the cited material), but not neglecting any older relevant studies. For cell-based studies, the keywords and MeSH terms used were: “tocopherol”, “tocotrienol”, “vitamin E” AND “anti-inflammatory”, “anticancer”, “metabolic” AND “cellular effects”, “pathway”, and “signaling.” The 21 most relevant papers were selected after eligibility analysis and cross-checking. For preclinical studies, the keywords and MeSH terms used were: “tocopherol”, “tocotrienol”, “vitamin E” AND “mice”, “rats” AND “anti-inflammatory effect”, “anticancer effect”, “antihyperlipidemic effect”, and “neuroprotective effect.” A total of 41 papers were selected after eligibility analysis and cross-checking. For clinical trials, the used the keywords and MeSH terms were: “tocopherol”, “tocotrienol”, “vitamin E” AND “clinical trial” AND “anti-inflammatory”, “neuroprotective”, “cardiovascular”, “metabolic”, “neurodegenerative” AND “effects”, and “disease.” A total of 70 papers were selected after eligibility analysis and cross-checking. Additionally, literature was reviewed in order to ascertain the key aspects regarding the intake, bioavailability, and metabolism of vitamin E; the keywords and MeSH terms used were: “tocopherol”, “tocotrienol”, “vitamin E” AND “dietary sources”, “daily intake”, “absorption”, “bioavailability”, and “metabolism”.

## 3. Structures, Dietary Sources, and Daily Intake

### 3.1. Structures

**Figure 1:**
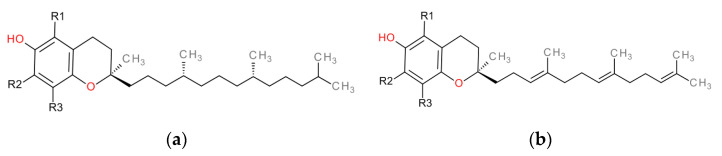
The general structure of tocopherols (TFs) (**a**) and tocotrienols (TTs) (**b**).

Natural forms of vitamin E include eight chemical forms that have a chroman ring system (2-methyl-6-hydroxychroman) as a basic structural unit and a side chain of 16C atoms. The vitamin E compound family include two subgroups: TFs and TTs, a shown in Figure 1. The TFs have a saturated side chain known as a phytanyl tail, while the TTs have an isoprenoid chain. In each group, there are four homologues (α-, β-, γ-, and δ-) that differ in number and in the position of the methyl substituents in the chroman ring Table 1 [13].

The functional groups present on the benzene ring are a phenolic hydroxyl and at least one methyl. The phenolic hydroxyl group is responsible for the antioxidant activity, and the number of methyl radicals influence their activity; thus, α-TF is the most active in the in the TF homologues series due to its three methyl groups, and δ-TF, with one methyl group, is the least active. The TF scaffold has three chiral centers—the C2 in the chroman ring and C4′ and C8′ in the side chain; the TT molecule only has the C2 in the chroman ring. All the natural TFs have a 2*R*, 4*R*, 8*R* (RRR) configuration, and the TTs have an R-configuration. The synthetic compounds are a mixture of 3R-stereoisomers, 2R-stereoisomers, and 2S-stereoisomers, named all-rac-α-TF. The biological activity and in vivo biopotencies are different between the TF stereoisomers, so it is important to make this distinction. The only forms retained in human plasma are the RRR-α-TF and the 2R-stereoisomers [14]. As RRR-α-TF accounts for about 90% of vitamin activity in human tissues, overall vitamin E activity is expressed as equivalents of it. The relative potency of α-, β-, γ-, and δ-TF is reported to be approximately 100:50:25:1 [15]. 

### 3.2. Dietary Sources

As a fat-soluble natural compound, vitamin E is found in plant-based oils, nuts, seeds, fruits, and vegetables. The proportion of the four TFs varies in different parts of the plant. In leaves, the most abundant is α-TF [16], while β-, γ-, and δ-TFs and TTs tend to predominate in seeds [17]. Additionally, the proportion of these vitamin E isomers substantially differs between the same plant varieties [18]. Moreover, the total amount of vitamin E in the product varies with the oil extraction method [19] or the cooking method [20]. 

The richest source of tocopherols is wheat germ oil, which specifically contains α-TF (1.36–1.37 mg/g) and β-TF (0.99–1.2 mg/g) [21]. Other major sources for vitamin E are: soybean seed oil (1.2 mg/g total TFs with 7% α-TF, 70% γ-TF, and 22% δ-TF), corn seed oil (1 mg/g total TF with 20% α-TF, 70% γ-TF, and 7% δ-TF), and sunflower seed oil (0.7 mg/g total TF with 96% α-TF, 4% γ-TF, and δ-TF) [17]. 

Vitamin E can also be found in: safflower oil, coconut oil [22], rapeseed oil [19], palm oil, olive oil, almonds [23], peanuts, pistachio, and walnuts [24]. Some fruits and vegetables also have significant amounts of vitamin E (more than 1 mg/100 g edible weight), such as avocado, blackberries, cranberries, kiwi, asparagus, broccoli, and spinach [20]. 

### 3.3. Daily Intake

There are two ways to express the doses and the amount of a liposoluble vitamins in a product: one based on quantity, with the measurement unit being the mg and one based on biological activity, with the measurement unit being the international unit (IU). Even though IUs are no longer recognized, they still can be found in some labelling [25]. In 2016, the American Food and Drug Administration (FDA) established that the label declaration should be in mg α-TF and required manufacturers to use these new labels starting from January 2020, but companies with annual sales of less than $10 million may continue to use the old labels that list vitamin E in IUs until January 2021 [26]. The conversion factors that manufactures have to use are presented in Table 2 [27].

In US, the Food and Nutrition Board (FNB) at the Institute of Medicine of The National Academies (formerly National Academy of Sciences) developed Dietary Reference Intakes (DRIs) that include estimated average requirement (EAR), recommended dietary allowance (RDA), adequate intake (AI), and tolerable upper intake level (UL). For vitamin E, the RDA refers to how much RRR-α-tocopherol (the only form that naturally occurs in food) is maintained in the blood and has biological activity, as well as to the 2R-stereoisomeric forms of α-tocopherol that occur in fortified foods and supplements; these values are detailed in Table 3 [28]. 

In the European Union (EU), the most recent scientific opinion of European Food Safety Authority (EFSA) was from 2015 and the only form of vitamin E that was considered was α-TF. The Panel on Dietetic Products, Nutrition and Allergies (NDA) considered that average requirements (ARs) and population reference intakes (PRIs) for vitamin E cannot be derived for adults, infants, and children, and it therefore defines AIs based on the observed intakes in healthy populations with no apparent α-tocopherol deficiency in the EU. The values for AIs are presented in Table 4 [29]. 

For pregnant or lactating women, the NDA considers that there is no evidence for an increased dietary α-TF requirement, and the same AI is set as for non-pregnant non-lactating women [29]. Overall, a daily intake of 12–15 mg/day of vitamin E, in normal healthy adults, is considered sufficient to provide adequate vitamin status. 

### 3.4. Bioavailability and Factors That Influence It 

Bioavailability is defined as “the degree and rate at which a substance is absorbed into a living system or is made available at the site of physiological activity” [30]. To determine this for vitamin E vitamers from various food sources, it is necessary to assess their absorption, transport, and distribution in the body. Regardless of which form is administered, the bioavailability of vitamin E can be influenced by numerous elements. The complexity of the involved metabolic processes and the multitude of available vitamin E forms justify the huge number of studies undertaken to establish the bioavailability and potency of vitamin E, as well as the influence of various factors on these parameters.

#### 3.4.1. Absorption, Distribution, and Metabolism

Dietary vitamin E (post-intestinal passage) is imbedded into lipoproteins. Phospholipid transfer protein (PLTP) is responsible for enriching lipoproteins with this liposoluble antioxidant for the exchange of vitamin E between HDL and the other lipoproteins, as well as for the normal distribution of vitamin E in tissues (brain and even spermatozoa) [31,32,33,34].

The RRR-α-TF is the preferred form secreted from the liver, distributed from plasma to tissues, and incorporated into VLDL/HDL; these phenomena are controlled by the α-TF transfer protein (α-TTP). The RRR-α-TF is also an important regulator of the metabolism and excretion of other vitamin E forms [35,36,37]. Interestingly, α-TF supplementation leads to a decrease of γ-TF plasma concentrations, due to the function of the hepatic transfer protein, responsible for preferentially secreting α-TF into plasma and for increasing γ-TF metabolism [38,39,40].

The metabolic interactions of vitamin E have been debated in the literature; for example, preclinical studies showed that increased α-TF concentrations induced a significant increase of CYP3A protein expression; since the CYP3A4 family is involved in the metabolism of a lot of drugs (>50%), there is a strong possibility that high doses of TFs would alter the metabolism of associated drugs [35,41,42,43,44]. 

Based on preclinical data, according to the Food and Nutrition Board, the UL for α-TF is 1000 mg (1100 IU for synthetic (all-rac) and 1500 IU for natural (RRR)), so its toxicity is considered to be low. Still, the risk of drug interaction is a constant concern. For example, in a double-blind trial including 160 patients, results showed that the HDL-increasing effect of simvastatin/niacin was reduced and the adverse effects were more frequent in subjects also receiving antioxidants (e.g., vitamin C, vitamin E, α-TF, β-carotene, and selenium) [45,46]. The Women’s Angiographic Vitamin and Estrogen (WAVE) trial included 423 postmenopausal women with at least one coronary stenosis at baseline coronary angiography, and it showed that all-cause mortality was increased in women on hormone replacement therapy who received antioxidant vitamins. This was probably due to the fact that TF stimulated CYP3A4 drug metabolism and decreased drug concentrations (for hormones and CVD drugs) [47]. 

#### 3.4.2. Natural vs. Synthetic

The established biopotency ratio between the natural form (RRR-stereoisomers) and all-rac (synthetic forms) is now 2:1, but the first ratio determined using the fetal rat resorption model was 1.36:1 [48]. Hoppe and Krennrich reviewed the bioavailability studies for natural and synthetic vitamin E, and they highlighted that they have different pharmacokinetics due to their different chemical composition. Another important remark was that studies using the competitive uptake method (with natural and synthetic forms concomitantly administered to the same individual) have a great value for comparing plasma kinetics but are unreliable for estimating potency.

Bioavailability can be a surrogate for measuring the potency of preparations containing identical active ingredients, but in the case of these compounds, the authors proved that the studies need to aim at measuring the potency of vitamin E in humans in vivo or ex vivo. They demonstrated that the RRR:all-rac ratio varied not only with the timepoint but also with the dosage between 1.99 and 1.51 [49].

Another way to study bioavailability is using a non-competitive uptake method: deuterium-labelled natural and synthetic vitamin E are administered on separate occasions to the same individual. Lodge used this approach to compare the plasma biokinetics of deuterated natural (RRR) and synthetic all-rac-tocopheryl acetate in smokers and non-smokers, and they demonstrated that the RRR:all-rac ratio was 1.3:1 in non-smokers and 0.9:1 in smokers [50]. Additionally, with the different biopotency ratios (which were similar with those derived from animal studies in the 80 s by Weiser et al. [48,51]), Lodge demonstrated that smokers and non-smokers handle natural and synthetic-TF differently and this can be a factor that contributes to inter-individual variation. 

#### 3.4.3. Dietary Factors 

Vitamin E, like all fat-soluble vitamins, needs dietary lipids for the formation of micelles, which are emulsified in the presence of bile salts, in order to be absorbed [50]. The questions in this case are: how much fat and what kind of fat?

Jeanes et al. compared the absorption of a stable-isotope-labelled vitamin E following meals with varying fat contents and sources: toast with butter (17.5 g fat), cereal with full-fat milk (17.5 g fat), cereal with semi-skimmed milk (2.7 g fat), and water (0 g fat). They found that there was a significant difference between high-fat and low-fat meals, with none between low-fat and water, and they reported a borderline difference between the two high-fat meals (*p* = 0.065). The discrepancy between vitamin E absorption following low-fat and high-fat meals demonstrates the need for certain amount of fat, with 2.7 g proving insufficient in influencing bioavailability in this case. Secondly, the difference between the two high-fat meals indicated that both the amount of fat and the food matrix are factors [52].

Another study used deuterium-labeled α-tocopheryl acetate-fortified apples to evaluate the influence of fat on vitamin E absorption. The apples were consumed at a breakfast containing 0%, 6%, or 21% kcal from fat in three sequential trials. The results confirmed that the presence of fat increased the absorption of vitamin E from 10 ± 4% (0% fat) to 20 ± 3% and 33 ± 5% in the 6% and 21% fat trials, respectively [53].

Vinson et al. studied the effect of *Aloe vera* liquid preparation consumption on the absorption of water- or fat-soluble vitamins and proved that absorption was slower and that vitamins were retained longer in plasma when given together with the *Aloe*-based preparations [54].

#### 3.4.4. Physiological and Pathological Factors

Each person is different, and that can also be observed in the case of vitamin E bioavailability. Several diseases, such as cystic fibrosis, short-bowel syndrome, chronic cholestatic hepatobiliary disease, Crohn’s disease, exocrine pancreatic insufficiency, and liver diseases, are associated with fat malabsorption and, consequently, with a vitamin E deficit [55].

Among physiological factors, age, sex, and genetic background are the most notable. A substance’s bioavailability can vary with age and sex, but those facts are already well-known, and RDAs are established accordingly. In contrast, the genetic background is specific for each person and can lead to important inter-individual variability. Some genetic diseases affect the absorption process of liposoluble vitamins. For example, abetalipoproteinemia is an autosomal-recessive disease that causes an error in lipoprotein production and transport [55]. Furthermore, the inter-individual variation of vitamin E bioavailability can be explained by a combination of single-nucleotide polymorphism (SNP) in genes involved in vitamin E metabolism. Desmarchelier et al. found that the ability to respond to vitamin E appears to be, at least in part, genetically determined [56]. They identified 32 SNPs in 13 genes and were able to validate a model that explained the variance of the vitamin E response based on triacylglycerol (TG) concentration with good predictive capacity (79%) [57].

#### 3.4.5. Technological Factors

Compared to dietary intake, vitamin E supplements offer a precise dose with a predefined profile. The incorporation of vitamin E in pharmaceutical formulations has some limitations due to its poor water-solubility, which limits its absorption in the gastrointestinal tract, and its sensitivity to oxygen, light, and temperature variations [58]. 

Several techniques can be used to obtain stable products with vitamin E, e.g., a self-emulsifying formulation was found to produce an increase in bioavailability between 210 and 410% compared with soft gelatin capsules under a fasted condition [59]. Encapsulation has shown promising results for protecting bioactive molecules against light, humidity, and oxygen, masking the taste and odor, and increasing the solubility and dissolution rates. 

There are many methods to obtain microcapsules, such as spray drying, freeze drying, complex coacervation, and emulsification [60]. Spray-freeze-drying is an unconventional freeze drying technique that includes three main process steps: atomization, freezing, and drying [61]. Parthasarathi et al. compared the bioavailability of vitamin E microcapsules obtained by the three different methods of spray freeze-drying, spray drying, and freeze-drying by using a whey protein isolate as an encapsulating agent. In male rats, spray freeze-dried microcapsules achieved a maximum vitamin E plasma concentration of 9.449 µg/mL at 3 h, whereas spray dried and freeze-dried microcapsules achieved 7.348 and 7.693 µg/mL, respectively [62].

The use of cyclodextrins to enhance the solubility of different compounds is well-known. For α-TF, a core-shell, bionanocomposite hydrogel based on β-cyclodextrin-soy soluble polysaccharide was used to obtain a sustained in vitro release (over 230 h) and a prolonged increase of plasma vitamin E levels over 12 h post-administration in vivo [63]. Another example is a γ-TT inclusion complex with γ-cyclodextrin, which improved the oral bioavailability of γ-TT [64]. 

## 4. Molecular and Cellular Mechanism of Action

The regulatory effects of TFs and TTs were tested using both cancerous (prostate, breast, HeLa, myeloid, adenocarcinoma, etc.) and non-malignant cell lines (neutrophils, macrophages, epithelial cells, etc.), with different study designs and experimental conditions. The most relevant studies we found regarding the purpose of this review are presented in Table 5.

In neutrophils, TFs and their metabolites (e.g., CEHC—2,7,8-trimethyl-2-(beta-carboxyethyl)-6-hydroxychroman) were found to regulate PKC signaling and the activities of NADPH and xanthine oxidase in a PMA-stimulated model [65]. Furthermore, they inhibited the generation of leukotriene B4 (LTB4), with no direct effect on 5-LOX (γ-TF, δ-TF, and γ-TT >> α-TF), while 13′-carboxychromanol, a long-chain metabolite of δ-TF, was a potent 5-LOX inhibitor. δ-TF prevented ionophore-induced intracellular calcium rise and ERK1/2 (extracellular signal-regulated kinase) activation [66].

In macrophages, TTs (especially δ-TT) proved to exert better anti-inflammatory effects than α-TF, reducing TNF-α, IL-1β, IL-6, PGE2, COX-2, and iNOS expression, as well as NF-κB activation, in LPS-stimulated models [67,68,69]. γ-TF and its metabolite, γ-CEHC (2,7,8-trimethyl-2-(beta-carboxyethyl)-6-hydroxychroman), decreased PGE2 synthesis via the inhibition of COX-2 activity—possibly a competitive inhibition mechanism, as the effects of α-TF were modest [70]. 

γ-TT reduced the TNF-α-induced activation of NF-κB and the expression of LPS-stimulated granulocyte-colony stimulating factor, upregulating C/EBPs [71]. δ-TT also decreases the TNF-α-induced activation of NF-κB (via TAK1 and A20 signaling) and the LPS-stimulated expression of IL-6 in a time- and dose-dependent manner [72]. TFs were able to prevent TNF-α-induced oxidative stress, increasing ICAM-1 an Cl-2 expression in intestinal epithelial cells (via redox and non-redox mechanisms), while their sulfide and disulfide derivates were even more active [73]. However, in fetal-derived intestinal cells, TFs enhanced NF-κB and Nrf2 signaling after an IFN-γ/PMA challenge, possibly contributing to a pro-inflammatory response [74]. γ-TT was found to decrease TNF-α-stimulated inducible and constitutive NF-κB activation [75], as well as STAT3 activity and its DNA binding activity in various cancer cell types (in contrast with γ-TF), which was found to be correlated with the inhibition of Src kinase and JAK1 and JAK2 kinases [76].

In an assessment of blood and endothelial cytotoxicity (TNF-α stimulation), TFs (especially δ-TF) proved useful in mitigating inflammation and angiogenesis [77], while in lung epithelial cells, TFs (α, γ, δ) and γ-TT decreased the IL-13/STAT6-stimulated expression of eotaxin-3 [78].

Several of the cellular pathways modulated by TFs and TTs have been highlighted in cancer cells studies. In melanoma cells, δ-TT-activated, ER stress-related pathways (activation of the PERK, IRE1α, and caspase-4 signaling) were found to result in pro-apoptotic activity [79]. In an esophageal epithelium cell carcinogenesis model, α-TF reduced cell proliferation and increased PPARγ expression, along the downstream PTEN tumor suppressor, acting as a PPARγ agonist [80]. 

Compared to α-TF, which had little effect in pancreatic cancer cells, δ-TT was efficient in augmenting gemcitabine activity, with a significant suppression of NF-κB activity and the expression of NF-κB transcriptional targets [81].

Several cellular pathways were reported to be regulated by various vitamin E forms in prostate cancer cells. α-TF reduced the TNF-α-stimulated expression of ICAM-1, VGEF, IL-6, and IL-8, as well as the activation of NF-κB and AP-1 [82]. δ-TF interfered with EGF signaling, resulting in the inhibition of the receptor tyrosine kinase-dependent activation of Akt [83]. Moreover, these are reports which suggest that CEHC metabolites of TFs are as effective as their precursors in inhibiting cell proliferation (via the specific downregulation of cyclin D1), with γ-derivates being the most efficient [84]. As for TTs, δ-TT was found to present cytotoxic/pro-apoptotic effects via ER stress, autophagy, and paraptosis pathways (the activation of JNK and p38) [85], as well as by reducing the expression of HIF-1α in a dose-dependent manner under hypoxic conditions [86].

In colon cancer cell lines, TFs were found to exhibit an anti-inflammatory effect and promoted apoptosis (especially δ-TF) in an IFNγ/PMA model [87], suppressing the activation of NF-κB (α-TF and γ-TF) and enhancing the Nrf2 pathway (δ-TF) [88]; their overall effect on antioxidant defense also seemed to be dependent on an elevation of cytoplasmic Ca^2+^ [89]. Moreover, γ-TF increased PPARγ mRNA and protein expression (more efficiently versus α-TF or troglitazone), with possibly important implications in inflammatory diseases [90].

TTs showed significant cytotoxic/pro-apoptotic and metabolism modulatory effects in breast cancer cell lines, including the downregulation of phosphorylated PI3K and GSK-3 cell survival proteins (β-TT), increased mitochondrial stress [91], ER stress via PERK and pIRE1α signaling, PARP cleavage and caspase-7 activation (γ-TT) [92], dose-dependent AMPK activation, Akt and mTOR inhibition (γ-TT), the reduction of glycolysis gene expression, glucose utilization, and ATP production (γ-TT) [93,94].

Furthermore, in HeLa cells, γ-TT was found to inhibit cell proliferation and promote apoptosis via the mitochondria-mediated intrinsic apoptotic pathway, increasing the Bax/Bcl-2 ratio, caspase-3 activation, and cleavage of PARP while also reducing the expression of PCNA [95].

These findings provided good evidence regarding the myriad of cellular pathways modulated by all vitamin E forms and their metabolites (e.g., CEHCs) or derivatives (e.g., disulfides), in both normal and cancer cells, gravitating towards reestablishing normal cell function. Table 5 includes details regarding the cell lines, design/treatment, and observed effects reported in the cited studies. TFs and TTs were shown to inhibit key enzymes in the arachidonic acid cascade, the generation of pro-inflammatory molecules, and the activation of the NF-κB pathway in pro-inflammatory environments and cancerous cells. Furthermore, they were found to exert cell cycle regulatory effects and to modulate responses to oxidative stress via in redox and non-redox mechanisms. Of all the studied molecules, δ-TF of the TFs and γ-TT of the TTs stood out as the most potent and versatile compounds with exciting potential for the chemoprevention and treatment of diseases with an important inflammatory component (e.g., cardiovascular or metabolic diseases).

## 5. Preclinical Reports

The protective actions of TFs and TTs are definitely a consequence of their direct scavenging effects of neutralizing reactive oxygen and nitrogen species, thus preventing oxidative cellular and DNA damage [96]. In addition to their scavenging role, vitamin E vitamers have a plethora of other effects. Further, animal-based models showed that the administration of TTs and TFs can modulate the activity of various enzymes and signaling pathways, such as MAPK, PI3K/Akt/mTOR, Jak/STAT, and NF-κB, thus acting as the underlying mechanisms of their reported anti-inflammatory, immuno-regulatory, neuroprotective, anti-proliferative, pro-apoptotic, anti-angiogenetic, and anti-metastatic effects [3,96]. 

### 5.1. Energy Homeostasis/Metabolism-Related Signaling

TTs and TFs profoundly influence metabolism, improving lipid profile and glycemic control in animal models of diabetes, hyperlipidemia, and non-alcoholic hepatic steatosis (NASH) [97,98,99,100,101,102,103,104]. These outcomes are due to their antioxidant and anti-inflammatory effects, as well as to their modulatory effect on various signaling pathways. TTs and TFs regulate AMPK (AMP-activated protein kinase) signaling; this enzyme monitors the AMP:ADP:ATP ratio in eukaryotic cells [105], and the AMPK/SIRT1/PGC1α pathway regulates insulin signaling in type 2 diabetic mice [97]. TT treatment was found to upregulate insulin receptor subunit-1 (IRS-1) and Akt levels, as well as to increase the translocation of GLUT4, enhancing insulin sensitivity and leading to a reduction of hyperglycemia in various animal models. An extensive review by Pang and Chin (2019) accurately explained the complex mechanism of metabolism modulation by TTs [100]. 

TFs, α-TT, γ-TT, and δ-TT were also reported to attenuate adipocyte enlargement, hepatic steatosis, and metabolic inflammation in the livers of obese and non-obese rats via hepatic PPARα/PPARγ activation [101]. Increasing hepatic PPARα expression and its downstream-regulated genes (*ACOX* and *CAT-1*) leads to upregulation of IκB expression, which inhibits the activation and nuclear translocation of the pro-inflammatory transcription factor NF-κB [98]. δ-TT reduced the TNF-α mRNA level in an animal model of hepatic steatosis in obese mice [99]. γ-TT administration was found to reduce the hepatic PPARγ expression. in an HFCS diet-induced of non-alcoholic steatosis, preventing the conversion of hepatocytes to adipocyte-like phenotypes [101]. TGFα signaling is also reduced by the administration of γ-TT, so the activation of hepatic stellate cells (HSCs), the hallmark of hepatic fibrosis, was inhibited [101]. Other beneficial effects were observed for a palm oil TT-rich fraction (mixture of several TTs and tocopherol isoforms) [102], γ-TT [103], δ-TT [99], and Annatto-extracted TTs [104] in steatosis animal models, including a reduction of blood triglycerides, total cholesterol, and low-density lipoprotein (LDL) levels—effects correlated with the downregulation of fatty acid biosynthesis proteins/enzymes such as fatty acid synthase (FAS), sterol regulatory element-binding protein-1/2 (Srebf1/2), stearoyl-CoA desaturase-1 (Scd1), acetyl-CoA carboxylase-1 (ACC1), HMG-CoA reductase, low-density lipoprotein receptor (LDLR), diglyceride acyltransferase (Dgat2), ad lipoprotein lipase (Lpl). 

### 5.2. Regulatory Effects on Inflammation Pathways

A vitamin E mixture rich in γ-TF and γ-TT was shown to reduce serum 8-isoprostane and PGE2, as well as to subsequently alleviate inflammation-related symptoms in murine models of dextran sulfate sodium (DSS)-induced colitis [106], in non-alcoholic steatosis induced by a high-fat diet [101], in an airway inflammation model caused by intranasal LPS in rats [107,108]. The modulatory effect of γ-TF on allergic inflammation seems to depend on the route of administration: the subcutaneous administration of γ-TF was associated with airway inflammation and abolished α-TF-exerted anti-inflammatory effects via PKCα activation, a phenomenon that must be taken into consideration in clinical settings [108]. On the other hand, a decrease in LTB4 was observed after the administration of a diet containing a 0.3% γ-TF-rich mixture [109]. 

The inhibition of NF-κB signaling is one of the main mechanisms underlying the anti-inflammatory effects reported for TTs and TFs, as it leads to the decreased synthesis of several pro-inflammatory molecules [110,111]. NF-κB represents a protein complex, its family comprising five transcription factors, that binds to consensus target sequences in various promoters and regulates gene expression after activation [112]. 

TTs and TFs were reported to stimulate SIRT-1 activity, which suppresses NF-κB activation through the deacetylation of its p65 subunit [113] and by significantly increasing the expression of IκBα (γ-TT) [101] and A20 (δ-TT as a result of modulating sphingolipids) [72]—natural inhibitors of NF-κB.

In alloxan-induced diabetic ICR mice, orally administered γ-TF (35 mg/kg) was found to downregulate the expression of genes for TNF-α, IL-1β, and CRP [113]. γ-TT nebulization reduced pro-inflammatory cytokines IL-6 and IL-8 in a lung injury ovine model caused by burn and smoke inhalation. The decrease in cytokine synthesis was associated with a reduction of the obstruction score and edema [114]. A dietary intake of a 0.05% α-TF or 0.05% γ-TF-rich mixture inhibited the colitis-associated elevation of pro-inflammatory IL-6 in the colon [115]. In chemically-induced diabetes models, γ-TF and a γ-TT-rich fraction (α-TF: 21.8%; γ-TF: 1.0%; α-TT: 23.4%; and γ-TT: 37.4%) dose-dependently reduced inflammation-related markers including NF-κB, MCP-1, IL-6, IL-1β, and TNF-α in skeletal muscle and plasma [97,101]. 

The NF-κB-mediated transcription of *pro-Il-1β*, *pro-Il-18*, and *Nlrp3* is essential for generating inflammasome components, including NOD-like receptor protein 3 (NLRP3) [116]. Inflammasomes are multiprotein cytosolic receptors that detect damage signals. They recruit adaptor protein ASC (apoptosis-associated speck-like protein containing a caspase recruitment domain) and pro-caspase-1. Caspase-1 becomes active and transforms pro-IL-1β and pro-IL-18 to active IL-1β and IL-18. Excessive inflammasome activation is associated with various chronic inflammatory diseases including multiple sclerosis, Alzheimer’s disease (AD), atherosclerosis, and age-related macular degeneration [117]. γ-TF and TT were reported to reduce NLRP3-inflammasome in a model of hyperglycemia-induced hepatic damage [116], as well as in two models of NASH—one induced by high-fat–high cholesterol diet and the other induced by colin-methionine deficient diet [101]. Additionally, vitamin E was reported to reduce other pro-inflammatory gene expressions, such as those of monocyte chemoattractant protein 1 (MCP-1) or Cd11c [101]. 

TFs and TTs inhibit the formation of pro-inflammatory eicosanoids mediated by COXs and 5-LOX. A review by Lewis et al. identified post-translational changes of the COX-2 structure as a mechanism for PGE2 decrease following the administration of vitamin E derivatives. The inhibition of COX-2 by γ- and δ-TT attenuated inflammation induced by UVB and γ irradiation [118,119]. Eicosanoids from COXs and 5-LOX pathways contribute to cancer and metastasis development [120], so, the TF/TT-mediated inhibitory effect on COX-2 and 5-LOX was associated with cancer chemoprevention in chemically-induced/moderate colitis-promoted colon carcinogenesis [121,122] and with decreased ventral prostate epithelial dysplasia in a prostate cancer model induced by N-methyl-N-nitrosourea in rats [123]. The animal studies investigating the anti-inflammatory effects of vitamin E derivatives are summarized in Table 6.

### 5.3. Anti-Proliferative and Pro-Apoptotic Pathways 

NF-κB pathway inhibition contributes to the pro-apoptotic effect of TTs and TFs, leading to a depletion of anti-apoptotic proteins (Bcl-2, Bcl-xL, and cFLIP) with an increase in the expression of caspases (−8, −9, and −3), pro-apoptotic protein Bax, and PARP1 (nuclear poly(ADP-ribose) polymerase 1) cleavage in pancreatic cancer tissues [81]. The inhibition of NF-κB by TFs and TTs was also associated with the inhibition of the epithelial–mesenchymal transition. δ-TT was found to significantly decrease vimentin, a marker of the mesenchymal phenotype, and to increase E-cadherin, a marker of the epithelial phenotype in genetic and xenograft models of pancreatic cancer. The activation of NF-κB allows epithelial cells to acquire migratory and invasive characteristics that facilitate distant metastasis [124,125]. Furthermore, a γ-TF-rich mixture of TFs was found to suppress the incidence of palpable tumors and maintained redox sensitive transcription factor Nrf2, as well as Nrf2-regulated antioxidant genes in a murine prostate cancer TRAMP model [126].

PI3K/Akt/mTOR inhibition contributes to the anti-proliferative, pro-apoptotic, and anti-angiogenic effects of TTs and TFs. Lowering PTEN/PI3K/Akt signaling by a diet rich in δ-TF, but not in α-TF, resulted in a lower (~40%) prostate adenocarcinoma multiplicity in a murine model of prostate cancer (Pten^p−/−^ mice). The authors suggested that Akt signaling was partly affected by the antioxidant activity of TFs, as excess of ROS are known to stimulate this cellular pathway [127]. Additionally, high concentrations of γ-TF were proven to recruit PHLPP phosphatases to dephosphorylate pAkt, leading to its inactivation and the inhibition of its downstream cascade [128]. TTs exerted significant anti-angiogenic activity and pro-apoptotic effects in endothelial cells associated with increased levels of IL-24 mRNA in BALB/c mice [129]. These results were confirmed by other authors [130], who reported the downregulation of VEGF and CD31 expression (markers of angiogenesis) following γ-TT administration, through the abrogation of Akt/mTOR pathway in an orthotopic mouse model.

The Ras-Raf-MEK-ERK signaling pathway regulates cellular proliferation, differentiation, and survival [131]. Husain et al. reported that the administration of δ-TT (200 mg/kg × 2/day) decreased pMEK, pERK, and pAkt expression in pancreatic tumors using a transgenic mouse model of pancreatic cancer [124]. pERK inhibition by TTs and TFs was found to be associated with an increased expression of cell cycle inhibitor proteins p21^Cip1^ and p27^kip−1^, suggesting a potential cell cycle arrest [124]. These results were further confirmed by Huang et al., who administered a mixture of TTs in a nude mouse xenograft model (using a VCaP human prostate tumor) and correlated the increase of p21 and p27 with the suppressed expression of histone deacetylases [132].

The activation of upstream MAP kinases, such as p-p38, by γ-TT protects against ER stress by decreasing the expression of ER-stress responsive genes like BiP and CHOP [101]. δ-TF was also shown to induce apoptosis via the activation of the ATF4/CHOP-DR5, thus inhibiting urothelial tumorigenesis in a UPII mutant Ha-ras transgenic mouse model [133].

JAK/STAT inhibition by TFs and TTs in tumors and adjacent tissues has been reported in colorectal cancer models [110,111]. STAT inhibition seems to be mediated by an increase in SHP-1 (Src homology region 2 domain-containing protein tyrosine phosphatase-1), interfering with c-Myc (proto-oncogene) and cyclin D1 degradation. These are modulators of cell cycle progression that are tightly regulated and involved in cell growth and proliferation, thus partly explaining the anti-proliferative effect of TFs and TTs. An δ-TT isoform was shown to selectively inhibit tumor progression and metastasis in transgenic mouse models of pancreatic ductal adenocarcinoma. More precisely, it selectively inhibited pancreatic ductal adenocarcinoma stem-like cells by inhibiting the viability, survival, self-renewal, and expression of Oct4 and Sox2 transcription factors [125]. 

Animal studies have indicated that TTs and TFs possess complex anti-cancer effects—an interplay between anti-proliferative, pro-apoptotic, anti-angiogenetic, and anti-metastatic effects (Table 7).

In conclusion, preclinical studies have been accordance with the results of the in vitro studies. They have highlighted the importance of the modulatory effect of TTs and TFs on various signaling pathways, resulting in anti-inflammatory, immuno-regulatory, neuroprotective, and anticancer effects. 

## 6. Effects in Humans Regarding Cardio-Metabolic Health

Many observational/epidemiological studies have pointed out the inverse correlation between cardiovascular disease and vitamin E intake (mainly α- and γ-TFs). Additionally, the cancer and neurodegeneration risks, as well as the comorbidities associated with ageing, seem to be in an inverted relationship with the liposoluble vitamin’s plasma levels. Most of these studies had design limitations, and the TF/TT sources should be regarded with caution, since monounsaturated and polyunsaturated fatty acids have been found in most of the vitamin E sources [138,139].

### 6.1. Effects in Healthy Volunteers

There have been some studies investigating the effects of tocopherols in healthy volunteers. For example, in a double-blind, randomized, placebo-controlled, crossover study, healthy subjects received a γ-TF enriched mixture and then were challenged by intranasal endotoxin (LPS), results showing that TFs counter-acted the LPS-induced IL-1β increase and reduced local inflammation [140,141]. Another study proved that 300 mg/day of γ-TF, but not 400 IU of α-TF every other day for six weeks, reduced intense exercise induced-platelet aggregation in healthy sedentary subjects [142]. Additionally, a mixture of TFs was more effective in reducing ADP-induced platelet aggregation compared to α-TF when administered to healthy subjects [143]. 

In a randomized, double-blind, placebo-controlled study, the impact of short term 500 mg γ-TF/day (seven days) on vascular endothelial function (VEF) was investigated in healthy subjects who quit smoking. Results proved that vitamin E supplementation in association with smoking cessation induced a more significant increase of flow-mediated dilatation (FMD) compared to the group that did not receive the vitamin E. Additionally, the TNF-α, myeloperoxidase (MPO) and malondialdehyde (MDA) levels decreased, even if the oxidized LDL and urinary F2-isoprostanes did not follow the same kind of trend, thus sustaining an improvement of the vascular function under the effect of vitamin E [144]. 

Other clinical studies suggested that vitamin E is correlated with a decrease of the cardiometabolic risk; for example, the Women’s Health Study proved that 600 IU of vitamin E (on alternate days) induced a significant decrease of cardiovascular mortality (24%) in healthy women and a 49% decrease in women over 65 years old [145]. 

Moreover, TT-rich products (400 mg of palm oil extracts) proved to have protective effects on healthy volunteers in a placebo-controlled clinical trial, leading to increases of tetanus toxoid antibody, IL-4, and IFNγ induced by a tetanus toxoid vaccine challenge, as well as decreasing IL-6 levels. The same type of TT-rich products positively influenced the CRP levels in healthy female subjects [146,147]. 

In a randomized, single-blind, crossover study including healthy non-smoking men, the effect of TFs (500 mg of γ-TF, 60 mg of α-TF, 170 mg of δ-TF, and 9 mg of β-TF) on the endothelial function and oxidative stress markers was investigated after a glucose tolerance test. Results showed that γ-TF was associated with a reduction of post-prandial MDA level, demonstrating its ability to prevent oxidative stress lesions on vascular function. Additionally, γ-TF improved vascular function by reducing the effects of hyperglycemia on the asymmetric dimethylarginine (ADMA)/arginine ratio, thus improving the bioavailability of NO, but no influence was observed regarding inflammatory markers [144,148]. 

However, one of the first reports concerning vitamin E was that this group of liposoluble molecules are necessary for the normal functioning of the reproductive function, initially known as the “anti-sterility factor” [149]. Numerous environmental and life-style factors can affect fertility, e.g., pollutants, smoking, alcohol/drug abuse, and diet. [150,151]. Vitamin E, along with other antioxidants such as vitamin C, vitamin A, and selenium, yield protective effects with better maternal and perinatal outcomes [152]. Low plasma α-TF levels were linked to poor pregnancy outcomes due to higher risks of infection, anemia, and retarded growth [153]. Decreased serum levels of vitamin E were reported in women suffering habitual abortion [154]. Supplementation was not recommended due to worries about possible side effects or unfavorable pregnancy outcomes. However, these fears were proven unfounded. For example, the administration of 400 IU vitamin/day from week 14 to birth had no significant effect on the development of pre-eclampsia [155]. A comprehensive view regarding the link between vitamin E and reproductive health, including both clinical and preclinical reports, was previously published [156].

### 6.2. Cardiometabolic Diseases 

The main pathological area of interest correlated with vitamin E has been, for a long time, cardiovascular disease; on this topic, results are quite controversial, but some reports stated that antioxidant vitamins (such as C and E) induce a reduction of intimal thickness of coronary and carotid arteries in hypercholesterolemic and heart transplant patients [157,158]. Still, literature data are not in complete agreement concerning the involvement of vitamin E in cardiovascular protection; for example, there have been reports stating that the intake of vitamin E-rich foods has the ability to improve cardiovascular function, but results have not been reproduced by vitamin E supplementations [159,160].

Meta-analysis data (14 trials with 597 included subjects) showed that supplementation with vitamins C and E does not induce improvements of endothelial function and pointed out an increased heterogeneity of the reported results [161]. On the other hand, results from 27 published studies including 742 patients attested that supplementation with vitamin E is correlated with an improvement of endothelial function; there was a negative correlation between the plasma vitamin E levels and the endothelial outcome, the effects being more significant for patients with TF plasma levels lower than 20 mM [161]. In addition the well-known antioxidant effects, potential mechanisms for these actions, in correlation with preclinical studies, include the ability of vitamin E to stimulate the activity of eNOS, thus increasing NO bioavailability (increased synthesis and reduced inactivation by ROS), as well as the inhibition of NF-κB signaling with a consequent decrease of inflammation at the endothelial level [162,163,164,165].

Administering α-TF 500 mg/day or a mixture of α-TF 75 mg/day and γ-TF 110 mg/day to type 2 diabetes patients in a double-blind, placebo-controlled study induced a reduction of plasma F2-isoprostane associated with an increase of the blood pressure, though with no impact on inflammatory markers [166,167]. Additionally, the association of vitamin E with vitamin A and zinc improved glycemic control and insulin secretion in type 2 diabetics [168].

Another cross-sectional study, including 582 adults with different glucose status, investigated the effects of TFs on TNF-α, showing a strong inverse correlation of non-α-TFs with TNF-α in prediabetes patients; this relationship was maintained in those with normal glucose tolerance, but not in diabetics. In the first class of patients (prediabetes), the reduction of inflammatory status was stronger in impaired fasting glucose (IFG) individuals and of lower significance in subjects characterized by impaired glucose tolerance (IGT) or with both IFG and IGT [169]. Additionally, in a randomized double-blind, placebo-controlled trial including 68 women with polycystic ovary syndrome (PCOS), the effect of omega-3 fatty acids associated with 400 IU/day vitamin E for 12 weeks on insulin resistance. Results showed that the co-supplementation of omega-3 and vitamin E significantly improved the indices of insulin resistance, total testosterone, and free testosterone, even if no effects were observed regarding fasting plasma glucose [170]. Another randomized, double-blind, placebo-controlled trial including 43 women with PCOS investigated the effects of 400 IU/day vitamin E for eight weeks on markers of endothelial function. The results showed that vitamin E induced beneficial outcomes regarding body weight, angiopoietin 1 (Ang-1), the Ang-1/Ang-2 ratio, and the VEGF level [171].

Clinical studies and meta-analyses were used to investigate the potential anti-inflammatory mechanism of TFs; doses below 400 IU/day were not found to have any effects on inflammatory markers, but an increase to 600–1200 IU/day significantly reduced the CRP, IL-6, and TNF-α levels [172,173,174]. A recent meta-analysis including 33 trials and 2102 individuals found that vitamin E supplementation significantly reduced CRP and, in high doses (of ≥ 700 mg/day), TNF-α levels [5]. Regarding vitamin E vitamers, α-TF proved the most beneficial in ameliorating low-grade inflammation [5]. Vitamin E intake was associated to a lower probability of serum CRP levels higher than 3 mg/L, with supplementation leading to their decrease regardless of baseline values [175,176,177]. Additionally, supplementing men and women with 700 IU/day vitamin E for one month induced a significant increase of liposoluble vitamin concentration in lipoproteins—threefold increase in vitamin E in HDL and twofold in LDL/VLDL. This increase was associated with a decrease of hsCRP [178]. However, caution is recommended, since Miller et al. concluded that doses over 400 IU/day might contribute to an increase of overall mortality [179]. Even if TFs are well-known antioxidant compounds, literature data have linked high dose vitamin E exposure to pro-oxidative effects rather than antioxidant ones, correlating this type of exposure to an increased CVD mortality [179]. Experimental data have shown that α- and γ-TFs induce dose-dependent, pro-oxidant effects on HDL; clinical studies have confirmed this pro-oxidant outcome in type 2 diabetes patients [180,181,182]. 

In a randomized, placebo-controlled, double-blind study, the effect of 800 mg/day of α-TF, 800 mg/day of γ-TF, or a combination of the two for six weeks was investigated in subjects with metabolic syndrome. The results showed that the combination of the two forms of vitamin E induced a reduction of CRP level, as well as a decrease of oxidative stress markers (urinary nitrotyrosine and lipid peroxides) [4,29].

In hemodialysis and end-stage renal disease patients, a γ-TF rich mixture induced a reduction of plasma CRP and IL-6, and it also reduced the risk of acute kidney injury [183,184]. Additionally, a pilot randomized, double-blind, placebo-controlled trial investigated the effects of 400 mg of a TT-rich vitamin E product for 12 months on the renal function of the supplementation of patients with stage 3 chronic kidney disease (CKD). Vitamin E improved kidney function, as revealed by serum creatinine, as well as estimated glomerular filtration rate [185]. 

### 6.3. Neurodegenerative Maladies

The beneficial effects of vitamin E intake were reported for more than cardiovascular and metabolic diseases; using vitamin E supplements for more than 10 years was found to induce a decrease of neurodegenerative disease related-mortality, α- and γ-TFs being the forms most associated with a slower rate of cognitive decline [186,187,188,189]. 

Clinical data showed that α-TF plasma levels are decreased in AD patients [190]. Moreover, an increase of TTs plasma levels is associated with an improvement of cognitive function [191]. In a cross-sectional study, including 168 patients with AD, total serum TTs were significantly decreased in AD patients compared to controls (118 vs. 91 mmol cholesterol; *p* < 0.05) [192]. γ-TF level was found to be clinically correlated with a lower β-amyloid formation, as well as with a decrease of the neurofibrillary tangle generation, suggesting the neuronal protective role of TF [188,189]. A prospective study including 232 patients with no AD diagnosis proved that, at a six years follow-up, subjects with high TTs plasma levels were at lower risk of developing AD [193]. In another prospective study including 140 Finnish older adults, the authors concluded that patients characterized at baseline by higher β- and γ-TT levels were less susceptible to develop AD (eight years follow-up); still, the size of the study was considered small [193]. The AddNeuroMed-Project, which evaluated the correlation of all plasma vitamin E forms and markers of vitamin E damage (α-tocopherylquinone and 5-nitro-γ-tocopherol) with mild cognitive impairment (MCI) and AD confirmed these results. This study also showed that MCI and AD cases had 85% lower odds to be in the highest tertile of total TFs and total vitamin E, and they were, respectively, 92% and 94% less likely to be in the highest tertile of total TTs than the lowest tertile. Moreover, both AD and MCI were strongly correlated with increased vitamin E damage markers [192].

There have been clinical reports stating that α-TF could reduce functional decline in mild cases of AD; for example, 2000 IU/day of α-TF in a randomized trial induced such an effect [194,195,196]. Additionally, vitamin E-rich foods were found to be correlated with a decreased risk of neurodegeneration; subjects in the group with 9 mg of vitamin E/day were 25% more susceptible to develop dementia compared to those with 18.5 mg of vitamin E/day [197]. These types of clinical effects of vitamin E are correlated with its antioxidant mechanism; for example, in the Cache County Study, antioxidant vitamins (C more than 500 mg/day and E more than 400 IU/day) were found to be correlated with a reduction of AD prevalence [198]. TFs might exert their beneficial effects in preventing neurodegeneration due to the antioxidant mechanism, as well as due to their ability to modulate acetylcholinesterase activity, since this enzyme is increased in AD—preclinical reports showed that vitamin E restores this enzyme the same way donepezil does [199]. 

However, not all results are in agreement; for example, a clinical study including 769 patients pointed out the lack of effect regarding the progression of AD in vitamin E treatment (2000 IU/day) compared to a placebo [200]. Another study showed no beneficial cognitive benefits when treating older women with 600 IU/day of α-TF acetate [201]. In 341 AD patients receiving either 2000 IU/day of vitamin E, selegiline, or a combination of the two showed a reduced functional deterioration, but there was an increase in total mortality in groups receiving vitamin E [202]. Regarding overall mortality, results have also been inconsequential; a meta-analysis including 135,967 patients aged 47–84 years reported that doses of 400 IU/day vitamin E or above induce small increases of mortality, but another study including 246,371 subjects pointed out that up to 5500 IU/day does not have any effect on mortality [179,203]. 

### 6.4. Anti-Aging Effects 

The general anti-ageing outcome of vitamin E supplementation was also investigated, since CVD, neurodegeneration, and metabolic imbalance are possibly associated with the ageing process. 

A randomized, double-blind clinical trial was performed including 64 subjects receiving for six months either a 74% TT vitamin E supplement (160 mg/day) or placebo; the total DNA damage was decreased by vitamin E, and the effect was greater in the subgroup of older subjects (>50 years old); these results suggested a possible general anti-ageing effect of vitamin E [191,204]. 

A review of published literature data analyzed the potential detrimental effects associated with vitamin E deficiency in order to establish the anti-ageing potential of these compounds [205]. Results showed that high intake and high plasma levels of α-TF correlate with a lower incidence of bone loss, reduced physical function, or frailty, especially after hip fracture; there is a strong association between the decrease of α-TF intake and the reduction of bone mineral density [206,207,208]. 

Additionally, patients in the highest vitamin E tertile were found to be less likely to develop frailty compared to those in the lowest vitamin E tertile, probably due the potential of vitamin E to modulate oxidative phosphorylation in muscle fibers. There have been reports that a high concentration of vitamin E is associated with a higher activity of creatine-kinase, thus contributing to an increased repair of skeletal muscle [209,210].

### 6.5. Cancer-Related Reports

Research regarding the association of vitamin E forms and cancer is also heterogenous, even if the anti-inflammatory and oxidative stress/DNA-damage-reducing mechanism of TFs could lead to an apparently clear conclusion. For example, α-TF was reported in experimental and preclinical studies to inhibit mechanisms involved in cancer progression (see Table 7); also, some epidemiological data showed an inverse association of this vitamin E form and cancer risk. On the other hand, the preventive effects from large randomized studies are rather disappointing. Surprisingly, the Selenium and Vitamin E Cancer Prevention Trial (SELECT) demonstrated that supplementing healthy men with 400 IU/day α-TF is associated with an increased risk of prostate cancer [138,211,212]. 

Interestingly, in a phase I trial, doses of δ-TF, ranging from 200 to 3200 mg/day, were concluded to be safe and effective (inducing apoptosis of malignant tissue) in patients with premalignant or malignant lesions of the pancreas [12]. 

TTs were also investigated for their anti-cancer potential in a placebo-controlled, double-blind study, where a TT-rich mixture was investigated in association with tamoxifen to evaluate the five year survival in women with early stage breast cancer; the results showed that the TT adjuvant did not improve breast cancer-specific survival rate versus tamoxifen-placebo controls, and a decrease in the risk of mortality due to breast cancer in the TT group versus with the tamoxifen-alone control group was registered [213]. 

Results obtained in clinical settings regarding the effects induced by vitamin E are quite variable, but should be taken into account. Most results have been based on anti-oxidant and anti-inflammatory mechanisms, also postulated by experimental and preclinical data. Positive outcomes were highlighted by some clinical trials and meta-analyses regarding the potential of vitamin E to improve redox and inflammatory status in healthy subjects, with interesting results obtained for smokers. Additionally, patients with diabetes and metabolic syndrome seem to benefit from increasing their intake of vitamin E-rich food products, leading to improved endothelial function and inflammatory status, even if these results have not been reproduced for supplementation with TTs/TFs and seem to vary between published studies. Interestingly, reports regarding anti-inflammatory potential are more significant for patients characterized by a low baseline level of vitamin E. α-TF and γ-TF have noteworthy effects in correlation with neurodegeneration, with clinical data showing a reduction of AD in patients supplemented with vitamin E. Trials regarding the anti-cancer effects of vitamin E have not been consistent, since some of the reports have pointed towards a pro-carcinogenic effect of these compounds. However, results obtained regarding cardiovascular and neurodegenerative risks have suggested vitamin E an interesting candidate for age-related pathology mitigation. 

## 7. Outlook and Conclusions

Vitamin E, with its eight vitamers, is best-known as the most important liposoluble antioxidant in the human body. However, its numerous regulatory effects regarding the modulation of cellular pathways, signal transduction, and gene expression affecting cell cycle and function were reported.

TFs and TTs, along with their metabolites (e.g., CEHCs) and derivatives (e.g., disulfides), modulate a myriad of cellular pathways, generating effects observed in both normal and cancer cells. Inclining towards re-establishing normal cell function, these compounds inhibit key enzymes in the arachidonic acid cascade (COX-2 and 5-LOX) and the generation of pro-inflammatory molecules (chemokine, interleukins, and prostaglandins), lowering the response to pro-inflammatory stimuli. Furthermore, they are able to inhibit NF-κB activation, leading to additional anti-inflammatory and cell cycle regulatory effects, as well as modulating antioxidant defense via in redox- and non-redox-related mechanisms. These in vitro, cell-based studies have pointed to δ-TF and γ-TT as the vitamers with the highest potential for the treatment of malignancies and cardio-metabolic diseases with an important inflammatory component.

Animal-based models showed that the administration of TTs and TFs can modulate the activity of various enzymes and signaling pathways, such as MAPK, PI3K/Akt/mTOR, Jak/STAT, and NF-κB, which constitute the foundation of their reported anti-inflammatory, immuno-regulatory, neuroprotective, anti-proliferative, pro-apoptotic, and anti-angiogenetic effects.

In clinical settings, the observed protective effects (or lack thereof) of vitamin E intake or supplementation varied greatly. Though caution should be exercised when extrapolating the beneficial effects reported in preclinical studies to humans, the therapeutic potential of TFs and TTs should not be disregarded. Vitamin E improves redox and inflammatory status in healthy individuals (smokers and non-smokers), diabetics, and subjects with metabolic syndrome. However, source and dosage greatly influence the observed effects. When using vitamin E, bioavailability seems to be a key factor in obtaining a desired outcome. The clinically-observed anti-cancer effects of vitamin E are inconsistent, with both pro- and anti-malignant ones being reported. However, this could be the result of different designs (dosage and duration of administration) and the great variability of malignancies. Numerous trials underlined vitamin E’s neuroprotective action, especially as a tool for prevention and as an adjuvant in the therapy of AD.

Ultimately, these is a strong body of literature that points to the great potential of vitamin E in the prevention and treatment of diseases with an inflammatory or malignant component, making vitamin E an interesting candidate for the mitigation of ageing-associated pathologies.

## Figures and Tables

**Table 1:** The structure of TFs and TTs.

Tocopherols	Tocotrienols	R1	R2	R3
α-tocopherol (α-TF)	α-tocotrienol (α-TT)	CH_3_	CH_3_	CH_3_
β-tocopherol (β-TF)	α-tocotrienol (β-TT)	CH_3_	H	CH_3_
γ-tocopherol (γ-TF)	α-tocotrienol (γ-TT)	H	CH_3_	CH_3_
δ-tocopherol (δ-TF)	α-tocotrienol (δ-TT)	H	H	CH_3_

**Table 2:** Conversion factors between different forms of vitamin E [27].

From	Conversion to Mg α-Tocopherol (Label Claim)
1 mg α-TF	1
1 mg RRR-α-TF	1
2 mg all-rac-α-TF	1
1 U.I. Vitamin E from natural sources (RRR-α-TF) including its ester forms (RRR-α-tocopheryl acetate and RRR-α-tocopheryl succinate)	0.67
1 U.I. Vitamin E from synthetic sources (all-rac-α-TF) including its ester forms (all-rac-α-tocopheryl acetate and all rac-α-tocopheryl succinate)	0.45

**Table 3:** Dietary Reference Intakes (DRIs) for vitamin E (α-TF) in the US (mg/day) [28].

Age		0–6 mo	7–12 mo	1–3 y	4–8 y	9–13 y	14–18 y	18 + y	Pregnancy	Lactation
**DRI (mg/day)**	EAR			5	6	9	12	12	12	16
	RDA	−	−	6	7	11	15	15	15	19
	AI	4	5							
	UL			200	300	600	800	1000	1000	1000

**Table 4:** The recommended adequate intakes (AIs) of α-TF (mg/day) in the EU [29].

Age	7–11 mo	1–<3 y	3–<10 y	10–<18		18 + y	
**AI**				boys	girls	men	women
	5 *	6	9	13	11	13	11

* derived by extrapolating upwards from the estimated α-TF intake in exclusively breast-fed infants aged 0–6 months and rounding.

**Table 5:** Molecular and cellular effect of TFs and TTs reported in in vitro cell-based studies.

Cell Line	Design/Treatment	Observed Effects	Reference
Human isolated neutrophils	PMA-stimulated neutrophils model: ▪ Pre-incubation with α-TF (10–50 μM), γ-TF (0.1–4 μM), δ-TF (0.1–4 μM), α-CEHC (0.05–5 μM), γ-CEHC (0.05–5 μM), or δ-CEHC (0.05–5 μM) for 30 min ▪ Stimulation with PMA (10^−7^ M) for 3 min	➢ Inhibition of the translocation and activation of PKC: CEHC > TF ➢ TFs, but not CEHCs, directly inhibit NADPH oxidase and xanthine oxidase	[65]
Human blood neutrophils or differentiated HL-60 cells	▪ Pre-incubation with α-, γ-, δ-TF, or γ-TT for 10 min (0–50 µM) or 13′-hydroxychroman (0–15 µM) for 15 min ▪ A23187/ionophore-stimulated (1–2.5 μM)	➢ γ-TF, δ-TF, and γ-TT: ↓ LTB4 (IC_50_ 5–20 μM), no direct effect on 5-LOX ➢ 13′-hydroxychroman: ↓ LTB4 (IC_50_ of 4–7 μM) and potently inhibited 5-LOX (IC_50_ of 0.5–1 μM) ➢ δ-TF: ↓ ionophore-induced intracellular calcium increase and calcium influx and the subsequent signaling including ERK1/2 phosphorylation ➢ δ-TF prevented ionophore-caused cytoplasmic membrane disruption, which may account for its blocking of calcium influx	[66]
Raw 264.7 macrophages	LPS-stimulated inflammation model: ▪ α-TF ▪ Incubation with TT-rich fraction, α-, δ-, and γ-TT versus α-TF, (10 µg/mL for all) ▪ Stimulated with LPS (10 ng/mL)	➢ TT-rich fraction and α-, δ-, and γ-TT: ↓ LPS-induced IL-6, NO ➢ α-TT: ↓ TNF-α ➢ TRF and α-, δ-TT: ↓ PGE2 ➢ TT-rich mix, as well as δ- and γ-TT: ↓ COX-2 gene expression	[67]
Murine peritoneal macrophages	LPS-stimulated inflammation model: ▪ Incubation with TT-rich fraction, α-TF, and α-TF-acetate (5–30 µg/mL) ▪ Stimulated with LPS (1 µg/mL)	➢ ↓ LPS-induced NO, PGE2, TNF-α, IFNγ, IL-1β, and IL-6 (TT-rich fraction > α-TF and α-TF acetate) ➢ ↓ NF-κB activation (TT-rich fraction > α-TF, α-TF acetate) ➢ TT-rich fraction (10 µg/mL): ↓ COX-2 and iNOS gene expression	[68]
Murine RAW 264.7 cells and peritoneal macrophages (PM, prepared from BALB/c mice)	▪ LPS-stimulated inflammation model: ▪ RAW 264.7 cells: α-TF, α-TT, γ-TT, or δ-TT (4, 8, and 16 μM) for 1 h, then stimulated with LPS (1 µg/well) for 4 h ▪ PM: LPS (10 ng/treatment), LPS and α-TF (25, 50, and 100 μM), and LPS and δ-TT (10, 20, and 40 μM)	➢ RAW 264.7 cells: TT determined significant and dose-dependent inhibition of TNF-α ➢ PM: low concentrations of δ-TT (10 and 20 μM) blocked LPS-induced gene expression of TNF-α, IL-1β, IL-6, and iNOS	[69]
Murine RAW264.7 macrophages Human epithelial cancer cells (A549)	▪ Pre-incubation with 0–50 μM α-TF or γ-TF for 8–14 h ▪ RAW264.7 macrophages: TF incubation and 0.1 μg/mL of LPS were introduced for 14 h ▪ A549 cells: TF incubation and 10 ng/mL IL-1β for 24 h	➢ γ-TF and γ-CEHC: ↓ PGE2 synthesis (in both cell lines—IC50 of 7.5/4 μM, respectively and ≈30 μM) ➢ α-TF slightly reduced (25%) PGE2 formation (50 μM) in macrophages but had no effect in epithelial cells ➢ Inhibition of COX-2 activity, possibly as competitive inhibitors of arachidonic acid ➢ γ-TF: suppression of iNOS expression in LPS-stimulated macrophages	[70]
Raw 264.7 macrophages	LPS-stimulated inflammation model: ▪ Pre-incubation for 14–16 h with α-TF (50 µM), γ-TF (10–50 µM), δ-TF (10–50 µM), and γ-TT (5–40 µM) ▪ Stimulated with LPS (0.1 μg/mL)	➢ γ-TT: ↓ LPS-induces IL-6 synthesis (via blocking NF-κB activation) ➢ γ-TT: ↓ LPS-stimulated granulocyte-colony stimulating factor ➢ γ-TT blocks LPS-induced the upregulation of C/EBPβ without affecting C/EBPδ	[71]
Raw 264.7 macrophages	TNF-α-induced NF-κB activation model: ▪ δ-TT (10 or 20 µM) ▪ TNF-α stimulation: 10 ng/mL for 5 min	δ-TT: ➢ ↓ TNF-α-induced activation of NF-κB and LPS-stimulated IL-6 (dose- and time-dependent). ➢ ↓ TNF-α-induced phosphorylation of TAK1—essential for NF-κB activation ➢ ↑ A20—inhibitor of NF-κB by modulating sphingolipid metabolism	[72]
Intestinal epithelial cells (HT29)	TNF-α-induced stress model: ▪ α-TF (5–100 µN) ▪ γ-TF (5–100 µN) ▪ Bis-δ-Toc sulfide (δ-Toc)2S (5–100 µN) ▪ Bis-δ-Toc disulfide (δ-Toc)2S2 (5–100 µN) ▪ Versus N-acetylcysteine (20 mM)	All tocopherol derivates: ➢ Prevented TNF-α-induced oxidative stress ➢ ↑ ICAM-1 and CI-2 expression ➢ (δ-Toc)2S and (δ-Toc)2S2 were more effective than α- and γ-Toc ➢ Mechanism: antioxidant properties (regulation of ICAM-1) and both redox and non-redox-dependent action in the TNF-α-induced Cl-2 expression.	[73]
Fetal-derived intestinal (FHs 74 Int) cells	▪ 24 h pre-treatment with α-, γ-, and δ-TF (1, 10, and 100 µM) ▪ IFNγ (4000 U/mL)/PMA(0.05 µg/mL) challenge	➢ ↑ IL-8 expression: δ-TF > γ-TF > α-TF ➢ ↑ Activation of NF-κB and Nrf2 signaling: δ-TF > γ-TF ➢ ↓ Glutamate-cysteine ligase: δ-TF > γ-TF	[74]
Human myeloid KBM-5 cells Human lung adenocarcinoma H1299 cells Human embryonic kidney A293 cells Human breast cancer MCF-7Human multiple myeloma (U266) Head and neck squamous cell carcinoma (SCC4) tumor cells	▪ Pre-incubation with 25 μM γ-TT for 12 h and then with 0.1 nM TNF-α for 30 min	➢ ↓ TNF-α-induced inducible and constitutive NF-κB activation ➢ Inhibition TAK1/TAB1-induced NF-κB-dependent gene expression	[75]
Human multiple myeloma (MM) cell lines U266, MM.1R, and MM.1S (dexamethasone-sensitive) and MIA PaCa-2, PC3, and DU-145 cells	▪ γ-TT (0–80 µM) for 0–8 h versus γ-TF (0–80 µM)	➢ γ-TT inhibits (dose- and time-dependently) constitutively active STAT3 and its DNA binding activity ➢ γ-TT downregulates IL-6-induced p-STAT3, constitutively active Src, JAK1, and JAK2 ➢ γ-TT induced the expression of SHP-1 in a dose-dependent manner ➢ STAT3 inhibition by γ-TT is not cell type-specific	[76]
Immortalized human dermal capillary cells (HMEC-1) and HMEC-1A (a subcloned population of pure lymphatic endothelial cells)	▪ HMEC-1 → human blood cytotoxicity—BEC model ▪ HMEC-1A → lymphatic endothelial cytotoxicity (LEC) ▪ Pre-incubation with α-, γ-, or δ-tocopherol at 10, 20, or 40 μM for 24 h ▪ TNF-α at a concentration of 20 ng/mL stimulation and incubation for 16 h	BEC: ➢ δ-TF, γ-TF: ↓ cell density ➢ γ-TF: ↓ invasiveness ➢ δ-TF: ↑ cell permeability (48 h) ➢ ↓ Capillary tube formation: α-TF (40 µM), γ-TF (40 µM), and δ-TF (40 µM) ➢ ↓ TNF-α-induced VCAM-1 expression: α-TF, γ-TF, and δ-TF (dose-dependently) ➢ LEC: ➢ γ-TF and α-TF (40 µM): ↓ invasiveness ➢ δ-TF: ↑ cell permeability (48 h) ➢ ↓ Capillary tube formation: α-TF (10 µM) and γ-TF (10–20 µM)	[77]
Human lung epithelial A549 cells	▪ Pre-incubation with α-TF (50 µM), γ-TF (10–50 µM), δ-TF (50 µM), and γ-TT (5–20) for 14–18 h or γ-CEHC/resveratrol for 1 h ▪ IL-13 (10 ng/mL) stimulation for 24 h	➢ ↓ IL-13/STAT6-induced expression of eotaxin-3: γ-TT (IC50 ~15 μM) > γ-TF, δ-TF (IC50 ~25–50 μM) > α-TF	[78]
Melanoma cell lines, BLM and A375	▪ δ-TT (5–20 μg/mL) for 24 or 48 h	➢ Pro-apoptotic effect on both cell lines ➢ Activation of the PERK, IRE1α, and caspase-4 ER stress-related branches.	[79]
Human normal esophageal epithelium cells Het-1A	NMBA-induced carcinogenesis model: ▪ NMBA (100 μM), α-TF (25, 50, and 100 μM), or their combination for 48–72 h	➢ ↓ Cell proliferation, ➢ ↑ Cell cycle G2-phase arrest and apoptosis ➢ ↑ Expression of PPARγ and its downstream tumor suppressor PTEN	[80]
Human pancreatic cancer cells (MiaPaCa-2 and AsPc-1)	For NF-κB activity assessment: ▪ Pre-incubation (72 h) with α-, β-, γ-, δ-TT, and α-, δ-TF (0.05 µM), δ-TT (0.05 µM), and gemcitabine (0.02 µM)	➢ ↓ Survival ➢ ↓ NF-κB activity: γ- and δ-TT (nuclear extract), β-, γ-, and δ-TF (cytosolic fraction) ➢ δ-TT, not α- or β-TT, suppressed NF-κB/p65 and phosphorylated the IkBα expression and downregulation of Bcl-xL ➢ α-TF and α-TT → no effect on NF-κB activity	[81]
Human prostate cancer cell lines (PC-3, DU-145, LNCaP, and CA-HPV-10)	TNF-α-induced stress model: ▪ α-TF (succinate salt at 15–20 µg/mL) with overnight incubation ▪ TNF-α stimulation: 10 ng/mL over 60 min for NF-κB and AP-1 activity and 18 h for IL-6, IL-8, and VEGF expression ▪ α-TF (succinate salt at 15–20 µg/mL) in a 3 h-incubation for adhesion assay	➢ ↓ NF-κB activity and ICAM-1 expression ➢ ↑ AP-1 activation ➢ ↓ IL-6, IL-8, and VEGF expression ➢ ↓ Cell adhesion	[82]
Prostate cancer cell line DU145	▪ α-, γ-, and δ-TF (5–40 µM) ▪ EGF or IGF for 2, 5, 10, 15, 20, and 30 min	➢ ↓↓ Phosphorylation of Akt ➢ δ-T ↓ EGF/IGF-induced activation of Akt (via the phosphorylation of Akt induced by PIK3 activation)	[83]
Prostate cancer cell line PC-3	▪ Incubation for 24 h with α- and γ-TF, α- and γ-CEHC, Trolox, and α-TF succinate (α-TS) at a concentration range of 0.1–50 µM	➢ ↓ Cell proliferation: γ-CEHC > γ-TF > α-TF > α-CEHC > Trolox > α-TF (γ-CEHC, γ-TF—maximal inhibition of ~10 µM) ➢ ↓ Cyclin D1 expression: both TFs and CEHCs ➢ γ-TF and γ-CEHC also interfere upstream cyclin D1	[84]
Castration-resistant prostate cancer cells (PC3 and DU145)	▪ δ-TT (5–20 μg/mL) for 24 h	➢ Cytotoxic/pro-apoptotic activity ➢ In PC3 cells via endoplasmic reticulum (ER) stress and autophagy pathways; ➢ In DU145 cells via ER stress pathway ➢ ↑ Phosphorylated JNK and p38 (both cell lines)	[85]
Prostate cancer PC3 stem-like cells	▪ Pre-incubation for 6 h under hypoxic condition ▪ Treatment with δ-TT at indicated doses for 24 h under hypoxia: δ-TT (0–40 μM)	➢ Dose-dependent cytotoxic effect ➢ ↓ HIF-1α	[86]
CaCO-2 and primary FHs 74 Int cells intestinal epithelial cell lines	▪ Peroxyl radical-induced membrane oxidation: cells were incubated with α-, γ-, δ-TF (1, 10, and 100 µM) for 24 h before labeled with DPPP ▪ Inflammatory response: pre-incubation with TF isoforms (1, 10, and 100 µM) for 24 h, followed by exposure to IFNγ (8000 U/mL) and PMA (0.1 mg/mL) for 24 h	➢ Antioxidant capacity: δ-TF > γ-TF > α-TF (CaCO-2 and FHs 74 Int cells) ➢ ↓ Inflammatory response in the IFNγ/PMA-induced inflammation (Caco-2 cells), ↑ IL-8 and PGE2 (FHs 74 Int cells) ➢ Apoptosis-mediated cytotoxicity: δ-TF > γ-TF > α-TF (not cytotoxic)	[87]
CaCO-2 cells	▪ IFNγ (8000 U/mL)/PMA (0.1 µg/mL)-induced inflammatory response model ▪ 24 h treatment with α-, γ-, and δ-TF (1, 10, and 100 µM)	➢ Suppression of IFNγ/PMA-induced NK-κB activation: α-TF> γ-TF >> δ-TF (ineffective) ➢ IFNγ/PMA-induced activation of Nrf2: δ-TF >> γ-TF > α-TF (ineffective) ➢ δ-TF: ↑ Nrf2 + ↓ GSH/GSSG ratio => pro-oxidant activity, lowered by ascorbic acid (with an additional ↓ IL-8).	[88]
CaCO-2 cells	▪ α-, γ-, and δ-TF (2.5–50 µm)	➢ Rapid increase in cytosolic calcium for all isomers ➢ Intracellular calcium elevation is necessary for the TF-induced antioxidant impact	[89]
SW 480 human colon cancer cell lines	▪ α-TF and γ-TF (5 or 10 μM) versus troglitazone (positive control at 100 μM) ▪ Incubation for 24 h for mRNA expression and 48 h for protein expression	➢ ↑ PPARγ mRNA (γ-TF >> α-TF) ➢ γ-TF increased PPARγ expression much more efficiently than α-TF or troglitazone	[90]
Breast adenocarcinoma cell lines MDA-MB-231 and MCF7	▪ β-TT and γ-TT (10–50 μM) incubated for 24 and 48 h	➢ Cytotoxic effects: β-TT > γ-TT (IC50 significantly higher) ➢ Mild G1 arrest on both cell lines ➢ Mitochondrial stress-mediated apoptotic response in MDA-MB-231 cells ➢ β-TT: downregulation of phosphorylated PI3K and GSK-3 cell survival proteins	[91]
MDA-MB 231 and MCF-7 breast cancer cells	▪ α-TT and γ-TT (10–40 μM) incubated for 48 and 72 h	γ-TT: ➢ ↑ Apoptosis via PARP cleavage and caspase-7 activation ➢ Activation of PERK and pIRE1α pathway to induce ER stress ➢ ATF3—molecular target for γ-TT	[92]
MDA-MB-231 and MCF-7 and breast cancer cells	▪ γ-TT (0–7 μM) over a 96 h treatment	➢ Dose-dependent ↑ AMPK → ↓ Akt activity ➢ Dose-dependent ↓ phosphorylated-FOXO3 (inactivated) ➢ ↓ Expression of genes associate with metabolic signaling and glycolysis	[93]
MCF-7 breast cancer cells	▪ γ-TT (0–10 μM) over a 96 h treatment	➢ Dose-responsive inhibition of mammary tumor cell growth ➢ ↓ Glucose use and expression of associated enzymes (hexokinase-II, phosphofructokinase, pyruvate kinase M2, and lactate dehydrogenase A), intracellular ATP production, and extracellular lactate excretion ➢ ↓ Phosphorylated (active) Akt, phosphorylated (active) mTOR, and c-Myc but not HIF-1α or GLUT-1 ➢ Result were significant for higher concentrations (6 and 8 µM)	[94]
HeLa cells	▪ γ-TT (15–60 μM) for 12, 24, and 48 h	➢ Dose- and time-dependently inhibited cell proliferation and induced apoptosis ➢ Arresting the cell cycle at the G0/G1 phase and increasing the Bax/Bcl-2 ratio, the activation of caspase-3 and caspase-9, and the cleavage of PARP ➢ Downregulated the expression of proliferative cell nuclear antigen (PCNA) and Ki-67 ➢ Promotion of the mitochondria-mediated intrinsic apoptotic pathway	[95]

TF—tocopherol; TT—tocotrienol; CEHC—carboxyethyl hydroxychroman; PMA—phorbol–myristate–acetate; PKC—protein kinase C; NADPH—nicotine-adenine-dinucleotide phosphate; LTB4—leukotriene B4; 5-LOX—5-lipooxygenase; ERK1/2—extracellular signal-regulated kinases 1/2; LPS—lipopolysaccharide; NO—nitric oxide; TNF-α—tumor necrosis factor α; PGE2—prostaglandin E2; COX-2—cyclooxygenase 2; IFNγ—interferon γ; IL—interleukin; NF-κB—nuclear factor kappa-light-chain-enhancer of activated B cells; iNOS—inducible nitric oxide synthase; C/EBP—CCAAT-enhancer binding protein; TAK1—transforming growth factor β-activated kinase 1; ICAM-1—intracellular adhesion molecule 1; CI-2—claudin-2; Nrf2—nuclear factor-erythroid 2-related factor 2; TAB1—TGF-beta activated kinase 1/MAP3K7 binding protein 1; STAT3—signal transducer and activator of transcription 3; p-STAT3—phosphorylated signal transducer and activator of transcription 3; Src—Proto-oncogene tyrosine-protein kinase Src; JAK 1/2—Janus kinases 1/2; SHP-1—Src homology 2-containing protein tyrosine phosphatase; VCAM-1—vascular cell adhesion molecule 1; STAT6—signal transducer and activator of transcription 6; PERK—protein kinase R-like ER kinase; IRE1α—inositol-requiring enzyme 1 α; pIRE1α—phosphorylated inositol-requiring enzyme 1 α; NMBA—N-nitrosomethylbenzylamine; PPAR—peroxisome proliferator-activated receptor; PTEN—phosphatase and tensin homolog; p65—nuclear factor NF-kappa-B p65 subunit; IkBα—nuclear factor of kappa light polypeptide gene enhancer in B-cells inhibitor, alpha; Bcl-xL—B-cell lymphoma extra-large protein; VEGF—vascular endothelial growth factor; AP-1—activator protein 1; IGF—insulin growth factor; EGF—epidermal growth factor; ER—endoplasmic reticulum; JNK—c-Jun N-terminal kinase; p38—p38 mitogen-activated protein kinase; HIF-1α—hypoxia-inducible factor 1α; DPPP—1,3-Bis(diphenylphosphino)propane; GSH—glutathione; GSSG—oxidized glutathione; PI3K—phosphoinositide 3-kinase; GSK-3—glycogen synthase kinase 3; PARP—poly (ADP-ribose) polymerase; ATF3—activating transcription factor 3; AMPK—AMP kinase; FOXO3—forkhead box O-3; mTOR—mammalian target of rapamycin; GLUT-1—glucose transporter 1; Myc—proto-oncogenes; Akt—protein kinase B; Bax—Bcl-2-like protein 4; Ki-67—marker of proliferation Ki-67.

**Table 6:** Selected in vivo studies on TFs’ and TTs’ anti-inflammatory effects.

Animal Model	Dosage	Duration of Administration	Measured Parameters	Conclusion	Reference
High-fat diet (HFD) induced hepatic steatosis in male C57BL/6 J mice	α-TF and γ-TF: 0.7 and 3.5 mg/kg/day (1:5 ratio)	12 weeks	α-TF and γ-TF: ➢ Decreases of serum triacylglycerols (56%) ➢ Downregulate inflammatory markers (TNF-α and IL-1β) ➢ Upregulates hepatic PPAR-α expression and its downstream-regulated genes (*ACOX* and *CAT-1*) ➢ Inhibits hepatic NF-κB activation	In an HFD-setting, a combination of α-TF and γ-TF ameliorated adipocyte enlargement, hepatic steatosis, and inflammation modulated via PPAR-α/NF-κB signaling.	[98]
High-fat (45%) diet containing cholesterol (0.2%) in C57BL/6 male mice	γ-TT 0.1% in diet	5 weeks	γ-TT: ➢ ↓ Diet-induced lipogenic gene expression: PPARγ, Srebp1c, Fas, DGAT2, Scd1, and Lpl ➢ ↓ Protein expression related to de novo lipogenesis: acetyl-CoA carboxylase and fatty acid synthase ➢ ↓ Pro-inflammatory gene expressions: MCP-1, Cd11c, TNF-α, NLPR3, and IL-1β ➢ ↓ ER stress marker: BiP, CHOP, p-JNK, p-eIF2α, and p-p38 ➢ ↑ IκBα expression ➢ ↓ Fibrosis-related gene expression of α-Sma, Timp1, TGF-β, and HDAC9	γ-TT attenuates hepatic TG accumulation by improving insulin sensitivity and delays progression to NASH by reducing ER stress/hepatic fibrosis axis activation.	[101]
Airway inflammation caused by intranasal LPS in male F344 rats	γ-TF at 30 mg/kg (oral gavage), daily and LPS intranasal challenge (0, 5, or 20 µg)	Prior (2 days before) and during LPS challenge	γ-TF: ➢ ↓ Neutrophil infiltration, BALF PGE2, secreted mucins, and pro-inflammatory intraepithelial cytokines ➢ ↑ IL-10 and IFNγ	Dietary γ-TF inhibited airway neutrophil recruitment and mucus hyperproduction.	[107]
Allergy airway inflammation and asthma models in ovalbumin-sensitized and challenged BALB/c mice	α-TF or γ-TF 100 mg/kg, s.c. injection	Prior to and during antigen challenge	γ-TF: ➢ ↑ IL-12, IFNγ, and IL-2 ➢ ↓ IL-5, IL-10, MIP-1a, and MCP-1	γ-TF, not α-TF, attenuated airway inflammation.	[108]
Alloxan induced diabetes in ICR mice—excisional wounds were made by biopsy punches	γ -TF (35 mg/kg) p.o. 5 times/week	2 weeks	γ-TF reduced: ➢ Inflammatory response-related proteins NF-κB, IL-1β, and TNF-α. ➢ Oxidative stress-related markers (modulating Nrf2 signaling and expression of NQO1, HO-1, MnSOD, CAT, and GPx) ➢ Apoptosis-related markers SIRT-1, PGC1-α, and p53	γ-TF administration prevented diabetes-induced delayed wound healing via the inhibition of NF-κB and the reduction of oxidative stress.	[113]
Chemically induced (DSS 2%) colitis in male BALB/c mice	α-TF or γ-TF-rich mix (γ-TF:δ-TF:α-TF, 58:22:11) 0.05% in diet (group A versus group B)	A. 4 week TF-supplementation and 1 week concomitant colitis inductionB. 1 week TF-administration and colitis induction	γ-TF-rich mix and α-TF: ➢ ↓ Colitis-associated elevation of pro-inflammatory IL-6 ➢ ↑ Occluding expression ➢ ↓ Elevation of circulating LBP, a surrogate marker of gut barrier dysfunction ➢ γ-TF-rich mix modulated the gut microbiota in mice with DSS-induced colitis but not in healthy animals	α-TF- and γ-TF-rich mix significantly reduced diarrhea and fecal bleeding in mice, with superior efficacy in the case of supplementation prior to colitis induction.	[115]
Alloxan induced diabetes in ICR mice	γ-TF (35 mg/kg) p.o.	3 weeks	γ-TF: ➢ ↓ 4-hydroxynonenal level ➢ ↓ Protein levels of NLRP3 inflammasome-related markers (pro-/caspase-1, pro-/IL-1β) ➢ ↓ TNF-α, MCP-1, iNOS, and COX-2 ➢ ↓ NF-κB ➢ ↑ Nrf2, NQO1, CAT, and GPx	γ-TF reduces fasting blood glucose levels, ameliorates hyperglycemia-induced hepatic damage, reduces lipid peroxidation and oxidative stress, and inhibits apoptosis.	[116]
γ irradiation CD2F1	δ-TT (400 mg/kg) s.c.	24 h before and 6 h after total body irradiation at 5 or 8.75 Gy/min	δ-TT: ➢ ↓ DNA-damage marker γ-H2AX foci ➢ ↑ mTOR and phosphorylation of its downstream effector 4EBP-1, with consecutive activation of mRNA translation regulator eIF4E and ribosomal protein S6	δ-TT protects mouse bone marrow and human CD34^+^ cells from radiation-induced damage through the ERK activation-associated mTOR survival pathways.	[118]
UVB-induced inflammation in HR-1 hairless mice	γ-TT-rich mix (2.3 mg/day) p.o. in corn oil	14 days	γ-TT: ➢ ↓ Expression of COX-2, IL-1β, IL-6, and MCP-1 ➢ ↓ p38, ERK, and JNK/SAPK activation	γ-TT attenuates UVB-induced inflammation and skin thickening by inhibiting several pro-inflammatory pathways.	[119]
Chemically induced (DSS 1.5–2%) colitis in male BALB/c mice	0.1% γ-TF or γ-TF-rich mix (45% γ-TF, 45% δ-TF, and 10% α-TF) in diet a week prior to DSS administration	43/62 days	γ-TF: ➢ ↓ Ki-67 and catenin β1 in the colon	An γ-TF-rich, but not γ-TF-rich mix, attenuated moderate colitis induced by one cycle of 1.5% DSS, while neither was protective to severe colitis induced by 3 cycles of 2.5% DSS.	[122]

H2AX—H2A histone family member X; mTOR—mammalian target of rapamycin; COX-2—cyclooxygenase 2; IL—interleukin; MCP-1—monocyte chemoattractant protein-1; p38—p38 mitogen-activated protein kinase; p-p38—phosphorylated p38; ERK—extracellular signal-regulated kinases; JNK/SAPK—c-Jun N-terminal kinases; p-JNK—phosphorylated JNK; PPAR—peroxisome proliferator-activated receptor; Srebp1c—sterol regulatory element-binding protein 1; Fas—apoptosis antigen 1; DGAT—diglyceride acyltransferase; Scd—stearoyl-CoA desaturase-1; Lpl—lipoprotein lipase; Cd11c—integrin alpha X chain protein; NLPR3—NOD-, LRR-, and pyrin domain-containing protein 3; BiP—binding immunoglobulin protein (ER chaperone GRP78); CHOP—C/EBP homologous protein; p-eIF2α—phosphorylated eukaryotic initiation factor 2; IκBα—nuclear factor of kappa light polypeptide gene enhancer in B-cells inhibitor, alpha; α-Sma—α-smooth muscle actin; Timp1—tissue inhibitors of metalloproteinase; TGF-β—transforming growth factor beta; HDAC9—histone deacetylase 9; BALF—bronchoalveolar lavage fluid; PGE2—prostaglandin E2; IFNγ—interferon γ; Ki-67—marker of proliferation Ki-67; DSS—dextran sulfate sodium; NF-κB—nuclear factor kappa-light-chain-enhancer of activated B cells; TNF-α—tumor necrosis factor α; NQO1—NAD(P)H dehydrogenase quinone 1; HO-1—heme oxygenase 1; MnSOD—manganese-dependent superoxide dismutase; CAT—catalase; GPx—glutathione peroxidase; LBP—lipopolysaccharide binding protein; iNOS—inducible nitric oxide synthase.

**Table 7:** Selected in vivo studies on TFs’ and TTs’ anticancer effects.

Animal Model	Dosage	Duration of Administration	Measured Parameters	Conclusion	Reference
Orthotopic xenograft model of human pancreatic ductal adenocarcinoma in mice NIH severe-combined immunodeficient (SCID) nude mice	α-TT, β-TT, γ-TT, and δ-TT: 200 mg/kg and 2/day	4 weeks	δ-TT: ➢ ↓ Anti-apoptotic proteins (Bcl-2, Bcl-xL, and cFLIP) ➢ ↑ Expression of caspases (-8, -9, and -3), expression of Bax, and PARP1 cleavage	δ-TT reduces the growth of pancreatic ductal adenocarcinoma by modulating NF-κB signaling.	[81]
Chemically induced (4-(methylnitrosamino)-1-(3-pyridyl)-1-butanone) lung tumor in A/J mice	0.3% γ-TF-rich mix (57% γ-TF, 24% δ-TF, 13% α-TF, and 1.5% β-TF) in diet	6 weeks	γ-TF-rich mix: ➢ ↓ 8-OH-dG, γ-H2AX, and nitrotyrosine in cancerous lesions ➢ ↑ Cleaved-caspase 3 in cancerous lesions	γ-TF-rich mix significantly reduced tumor volume and tumor weight.	[109]
Xenograft tumor growth (human lung cancer H1299 cells) in NCr-nu/nu mice		6 weeks		0.3% γ-TF-rich mix in diet significantly lowered the tumor multiplicity.	
Chemically induced (2-amino-1-methyl-6-phenylimidazo (4,5-b) pyridine) prostatic cancer in CYP1A-humanized mice (PhIP)	0.3% γ-TF-rich mix in diet (mixture of 56.8% γ-TF, 24.3% δ-TF, 13.0% α-TF and 1.5% β-TF) versus 0.2% δ-TF, γ-TF, or α-TF in diet	41 weeks	γ-TF-rich mix and δ-TF: ➢ ↓ 8-OH-dG, COX-2, nitrotyrosine, Ki-67, and p-AKT in prostatic lesions. ➢ ↑ PTEN and Nrf2 in prostatic lesions	γ-TF-rich mix and δ-TF significantly inhibited the development and severity of mouse prostatic intraepithelial neoplasia, being more effective than γ-TF or α-TF.	[110]
Nude mouse xenograft model of human colorectal cancer	100 mg/kg of γ-TT 5 times/week	2 weeks	γ-TT: ➢ ↓ Ki-67, cyclin D1, MMP-9, CXCR4, NF-κB/p65, and VEGF in tumor tissue	γ-TT reduced tumor growth and enhanced the antitumor efficacy of capecitabine, possibly by inhibiting NF-κB signaling. It induced apoptosis, inhibited colony formation, and suppressed key regulators of cell survival, cell proliferation, invasion, angiogenesis, and metastasis.	[111]
Orthotopic xenograft model of human pancreatic ductal adenocarcinoma in athymic mice	200 mg/kg of δ-TT 2/day	4 weeks	δ-TT: ➢ ↓ Ki-67 and inhibited expression levels of the stem cell transcription factors Nanog, Oct4, and Sox2 in cancerous lesions ➢ ↓ Notch1 receptor and KRAS downstream signaling factors pAkt and pERK in cancerous lesions ➢ ↑ E-cadherin expression in tumor tissue ➢ ↓ Expression of N-cadherin and vimentin in in tumor tissue	δ-TT reduces the growth of pancreatic ductal adenocarcinoma, inhibits pancreatic cancer stem-like cells, and prevents pancreatic cancer metastasis by reducing epithelial-to-mesenchymal transition.	[125]
Genetic: Ptenp−/− mice	0.2% δ-TF or α-TF supplemented in diet	34 or 28 weeks	δ-TF (not α-TF): ➢ ↓ pAkt ➢ ↓ Ki-67 ➢ ↑ Cleaved-caspase 3 in prostatic lesions	0.2% δ-TF, but not α-TF, diet increased apoptosis and reduced Akt activation and cell proliferation.	[127]
Orthotopic human colon cancer mouse model (HCCLM3) BALB/c nude mice	3.25 mg/day of γ-TT 5 days/week	5 weeks	γ-TT: ➢ ↓ Ki-67, VEGF, and CD31 in cancerous lesions ➢ ↑ Cleaved-caspase 3 in cancerous lesions	γ-TT reduces the tumor growth, and the tumor-induced angiogenesis by inhibiting AKT/mTOR pathway.	[130]
Genetic: UPII mutant Ha-ras transgenic mice	δ-TF 0.2% supplemented in diet	150 days	δ-TF: ➢ ↑ Expression of ER stress sensors PERK and IRE1α, as well as the downstream components BiP (GRP78), ATF4, and CHOP.	0.2% δ-TF diet had an antiproliferative effect and induced apoptosis via the activation of the ATF4/CHOP-DR5 pathway.	[133]
Chemically induced (estrogen) mammary hyperplasia in ACI rats	0.3% γ-TF-rich mix in diet (mixture of 56.1% γ-TF, 22.3% δ-TF, 11.5% α-TF, and 1.2% β-TF)	14 days	γ-TF-rich mix: ➢ ↓ 8-OH-dG and nitrotyrosine in hyperplastic mammary cells ➢ ↑ mRNA levels of Nrf2, SOD, CAT, and GPx in hyperplastic mammary cells ➢ ↓ Serum 8-isoprostane	γ-TF-rich mix exerted cytoprotective action and prevented estrogen-induced mammary hyperplasia.	[134]
Chemically induced colon cancer (azoxymethane and DDS) in C57BL/6 mice	0.1% mixed TTs and TFs in diet (>65% TTs) versus 1% DeltaGold/0.1% in diet (90% δ-TT and 10% γ-TT)	70 days	δ-TT: ➢ ↓ COX-2 protein levels in colorectal mucosa	δ-TT prevented colorectal cancer by inducing apoptosis and blocking the COX-2/PGE2 pathway that stimulates tumor–stromal interactions in colon cancer.	[135]
Chemically induced (azoxymethane) induced colon carcinogenesis in F344 Rats	0.2% δ-TF, γ-TF, or α-TF in diet	9 weeks	δ-TF treatment: ➢ ↓ The levels of 4-hydroxynonenal, nitrotyrosine, and the expression of cyclin D1 (colon) ➢ maintained the expression of PPARγ (colon) ➢ ↓ The serum levels of PGE2 and 8-isoprostane	δ-TF treatment showed the strongest inhibitory effect, decreasing the numbers of aberrant crypt foci and colon carcinogenesis.	[136]
Xenograft tumor growth (human lung cancer H1299 cells) in NCr-nu/nu mice	0.17% or 0.3% α-TF, δ-TF, γ-TF, or γ-TF-rich mix at diet	49 days	δ-TF and γ-TF-rich mix: ➢ ↓ 8-OH-dG, γ-H2AX, and nitrotyrosine in cancerous lesions ➢ ↑ Cleaved-caspase 3 in cancerous lesions	Growth inhibition effectiveness: δ-TF 0.3% > γ-TF-rich mix 0.3% > γ-TF 0.3% = δ-TF 0.17%> γ-TF-rich mix 0.17% = γ-TF 0.3% > α-TF 0.17% > α-TF 0.3%, with no significant differences versus control for α-TF.	[137]

8-OH-dG—8-oxo-deoxyguanosine; H2AX—H2A histone family member X; Ki-67—marker of proliferation Ki-67; p-Akt—phosphorylated protein kinase B; PTEN—phosphatase and tensin homolog; Nrf2—nuclear factor erythroid 2-related factor 2; COX-2—cyclooxygenase 2; MMP-9—matrix metallopeptidase 9; CXCR4—C-X-C chemokine receptor type 4; p65—transcription factor p65 (nuclear factor NF-kappa-B p65 subunit); VEGF—vascular endothelial growth factor; Bcl-2—B-cell lymphoma 2 protein; Bcl-xL—B-cell lymphoma-extra-large protein; cFLIP—CASP8 and FADD-like apoptosis regulator; PARP1—poly (ADP-ribose) polymerase 1 (PARP-1); Nanog—homeobox protein NANOG; Oct4—octamer-binding transcription factor 4; Sox2—sex determining region Y)-box 2; Notch1—notch homolog 1, translocation-associated; KRAS—Kirsten rat sarcoma viral oncogene homolog; NF-κB—nuclear factor kappa-light-chain-enhancer of activated B cells; pERK—phosphorylated extracellular signal-regulated kinase; CD31—cluster of differentiation 31 (platelet endothelial cell adhesion molecule); PERK—protein kinase R (PKR)-like endoplasmic reticulum kinase; IRE1α—inositol-requiring enzyme 1 α; BiP—binding immunoglobulin protein (ER chaperone GRP78); ATF4—activating transcription factor 4 (tax-responsive enhancer element B67); CHOP—C/EBP homologous protein; SOD—superoxide dismutase; CAT—catalase; GPx—glutathione peroxidase; PPAR—peroxisome proliferator-activated receptor; PGE2—prostaglandin E2.

## References

[1] Liu J.J., Green P., John Mann J., Rapoport S.I., Sublette M.E. (2015). Pathways of polyunsaturated fatty acid utilization: Implications for brain function in neuropsychiatric health and disease. Brain Res..

[2] Zingg J.M. (2019). Vitamin E: Regulatory Role on Signal Transduction. IUBMB Life.

[3] Lewis E.D., Meydani S.N., Wu D. (2019). Regulatory role of vitamin E in the immune system and inflammation. IUBMB Life.

[4] Chen Y., Varghese Z., Ruan X.Z. (2014). The molecular pathogenic role of inflammatory stress in dysregulation of lipid homeostasis and hepatic steatosis. Genes Dis..

[5] Asbaghi O., Sadeghian M., Nazarian B., Sarreshtedari M., Mozaffari-Khosravi H., Maleki V., Alizadeh M., Shokri A., Sadeghi O. (2020). The effect of vitamin E supplementation on selected inflammatory biomarkers in adults: A systematic review and meta-analysis of randomized clinical trials. Sci. Rep..

[6] Leon-Pedroza J.I., Gonzalez-Tapia L.A., del Olmo-Gil E., Castellanos-Rodriguez D., Escobedo G., CGonzalez-Chavez A. (2015). Low-grade systemic inflammation and the development of metabolic diseases: From the molecular evidence to the clinical practice. Cirugía y Cir..

[7] Minihane A.M., Vinoy S., Russell W.R., Baka A., Roche H.M., Tuohy K.M., Teeling J.L., Blaak E.E., Fenech M., Vauzour D. (2015). Low-grade inflammation, diet composition and health: Current research evidence and its translation. Br. J. Nutr..

[8] Ungurianu A., Margina D., Gradinaru D., Bacanu C., Ilie M., Tsitsimpikou C., Tsarouhas K., Spandidos D.A., Tsatsakis A.M. (2017). Lipoprotein redox status evaluation as a marker of cardiovascular disease risk in patients with inflammatory disease. Mol. Med. Rep..

[9] Ungurianu A., Seremet O., Gagniuc E., Olaru O.T., Gutu C., Gradinaru D., Ionescu-Tirgoviste C., Margina D., Danciulescu-Miulescu R. (2019). Preclinical and clinical results regarding the effects of a plant-based antidiabetic formulation versus well established antidiabetic molecules. Pharmacol. Res..

[10] Cheng P., Wang L., Ning S., Liu Z., Lin H., Chen S., Zhu J. (2018). Vitamin E intake and risk of stroke: A meta-analysis. Br. J. Nutr..

[11] Amanullah I., Khan Y.H., Anwar I., Gulzar A., Mallhi T.H., Raja A.A. (2019). Effect of vitamin E in non-alcoholic fatty liver disease: A systematic review and meta-analysis of randomised controlled trials. Postgrad. Med. J..

[12] Springett G.M., Husain K., Neuger A., Centeno B., Chen D.T., Hutchinson T.Z., Lush R.M., Sebti S., Malafa M.P. (2015). A Phase I Safety, Pharmacokinetic, and Pharmacodynamic Presurgical Trial of Vitamin E delta-tocotrienol in Patients with Pancreatic Ductal Neoplasia. EBioMedicine.

[13] Szymańska R., Nowicka B., Trela A., Kruk J. (2019). Vitamin E: Structure and forms. Molecular Nutrition: Vitamins.

[14] Kamal-Eldin A., Appelqvist L.Å. (1996). The chemistry and antioxidant properties of tocopherols and tocotrienols. Lipids.

[15] Scientific Committee on Food (2003). Opinion of the Scientific Committee on Food on the Tolerable Upper Intake Level of Vitamin E.

[16] Munné-Bosch S. (2005). The role of α-tocopherol in plant stress tolerance. Proc. J. Plant Physiol..

[17] DellaPenna D. (2005). A decade of progress in understanding vitamin E synthesis in plants. Proc. J. Plant Physiol..

[18] Zhang G.Y., Liu R.R., Zhang P., Xu Y., Zhu J., Gu M.H., Liang G.H., Liu Q.Q. (2012). Variation and Distribution of Vitamin E and Composition in Seeds Among Different Rice Varieties. Acta Agron. Sin..

[19] Chew S.C. (2020). Cold-pressed rapeseed (Brassica napus) oil: Chemistry and functionality. Food Res. Int..

[20] Chun J., Lee J., Ye L., Exler J., Eitenmiller R.R. (2006). Tocopherol and tocotrienol contents of raw and processed fruits and vegetables in the United States diet. J. Food Compos. Anal..

[21] Dunford N.T. (2009). Wheat Germ Oil. Gourmet and Health-Promoting Specialty Oils.

[22] Masterjohn C. (2007). The Anti-Inflammatory Properties of Safflower Oil and Coconut Oil May be Mediated by Their Respective Concentrations of Vitamin E. J. Am. Coll. Cardiol..

[23] Radcliffe J.D., Hernandez L.M. (2005). The Vitamin E Content of a Variety of Foods Made Exclusively from Almonds or Containing Almonds. J. Am. Diet. Assoc..

[24] Bonku R., Yu J. (2020). Health aspects of peanuts as an outcome of its chemical composition. Food Sci. Hum. Wellness.

[25] Institute of Medicine U.S. (2000). Vitamin E. https://ods.od.nih.gov/factsheets/VitaminE-HealthProfessional/.

[26] FDA (2017). Food Labeling: Revision of the Nutrition and Supplement Facts Labels and Serving Sizes of Foods That Can Reasonably Be ConsuMed. at One Eating Occasion; Dual-Column Labeling; Updating, Modifying, and Establishing Certain Reference Amounts Customarily Consumed; Serving Size for Breath Mints; and Technical Amendments; Proposed Extension of Compliance Dates. https://www.govinfo.gov/content/pkg/FR-2019-12-31/pdf/2019-27868.pdf.

[27] FDA (2019). Converting Units of Measure for Folate, Niacin, and Vitamins A, D, and E on the Nutrition and Supplement Facts Labels: Guidance for Industry. https://www.fda.gov/media/129863/download.

[28] (2006). Dietary Reference Intakes.

[29] EFSA (2015). Scientific Opinion on Dietary Reference Values for vitamin E as α-tocopherol. EFSA J..

[30] Bioavailability|Definition of Bioavailability by Merriam-Webster. https://www.merriam-webster.com/dictionary/bioavailability.

[31] Desrumaux C., Risold P.Y., Schroeder H., Deckert V., Masson D., Athias A., Laplanche H., Le Guern N., Blache D., Jiang X.C. (2005). Phospholipid transfer protein (PLTP) deficiency reduces brain vitamin E content and increases anxiety in mice. FASEB J..

[32] Drouineaud V., Lagrost L., Klein A., Desrumaux C., Le Guern N., Athias A., Menetrier F., Moiroux P., Sagot P., Jimenez C. (2006). Phospholipid transfer protein deficiency reduces sperm motility and impairs fertility of mouse males. FASEB J..

[33] Jiang X.C., Tall A.R., Qin S., Lin M., Schneider M., Lalanne F., Deckert V., Desrumaux C., Athias A., Witztum J.L. (2002). Phospholipid transferprotein deficiency protects circulating lipoproteins from oxidation due to the enhancedaccumulation of vitamin E. J. Biol. Chem..

[34] Kostner G.M., Oettl K., Jauhiainen M., Ehnholm C., Esterbauer H., Dieplinger H. (1995). Human plasma phospholipid transfer protein accelerates exchange/transfer ofa-tocopherol between lipoproteins and cells. Biochem. J..

[35] Panagabko C., Morley S., Hernandez M., Cassolato P., Gordon H., Parsons R., Manor D., Atkinson J. (2003). Ligand specificity in the CRAL-TRIO protein family. Biochemistry.

[36] Brigelius-Flohe’ R., Traber M.G. (1999). Vitamin E: Function and metabolism. FASEB J..

[37] Mustacich D.J., Bruno R.S., Traber M.G. (2007). Vitamin E. Vitam. Horm..

[38] Devaraj S., Leonard S., Traber M.G., Jialal I. (2008). Gamma-tocopherol supplementation alone and in combination with alpha-tocopherol alters biomarkers of oxidative stress and inflammation in subjects with metabolic syndrome. Free Radic. Biol. Med..

[39] Handelman G.J., Machlin L.J., Fitch K., Weiter J.J., Dratz E.A. (1985). Oral alpha-tocopherol supplements decrease plasma gamma-tocopherol levels in humans. J. Nutr..

[40] Sundl I., Resch U., Bergmann A.R., Roob J.M., Winklhofer-Roob B.M. (2004). The decrease in gamma-tocopherol in plasma and lipoprotein fractions levels off within two days of vitamin E supplementation. Ann. N. Y. Acad. Sci..

[41] Kluth D., Landes N., Pfluger P., Muller-Schmehl K., Weiss K., Bumke-Vogt C., Ristow M., Brigelius-Flohe R. (2005). Modulation of Cyp3a11 mRNA expression by alpha-tocopherol but not gamma-tocotrienol in mice. Free Radic. Biol. Med..

[42] Kliewer S.A., Goodwin B., Willson T.M. (2002). The nuclear pregnane X receptor: A key regulator of xenobiotic metabolism. Endocr. Rev..

[43] Mustacich D.J., Gohil K., Bruno R.S., Yan M., Leonard S.W., Ho E., Cross C.E., Traber M.G. (2009). Alpha-tocopherol modulates genes involved in hepatic xenobiotic pathways in mice. J. Nutr. Biochem..

[44] Traber M.G., Siddens L.K., Leonard S.W., Schock B., Gohil K., Krueger S.K., Cross C.E., Williams D.E. (2005). Alpha-tocopherol modulates Cyp3a expression, increases gamma-CEHC production, and limits tissue gamma-tocopherol accumulation in mice fed high gamma-tocopherol diets. Free Radic. Biol. Med..

[45] Brown B.G., Zhao X.Q., Chait A., Fisher L.D., Cheung M.C., Morse J.S., Dowdy A.A., Marino E.K., Bolson E.L., Alaupovic P. (2001). Simvastatin and niacin, antioxidant vitamins, or the combination for the prevention of coronary disease. N. Engl. J. Med..

[46] Cheung M.C., Zhao X.Q., Chait A., Albers J.J., Brown B.G. (2001). Antioxidant supplements block the response of HDL to simvastatin-niacin therapy in patients with coronary artery disease and low HDL. Arterioscler. Thromb. Vasc. Biol..

[47] Waters D.D., Alderman E.L., Hsia J., Howard B.V., Cobb F.R., Rogers W.J., Ouyang P., Thompson P., Tardif J.C., Higginson L. (2002). Effects of hormone replacement therapy and antioxidant vitamin supplements on coronary atherosclerosis in postmenopausal women: A randomized controlled trial. JAMA.

[48] Weiser H., Vecchi M. (1982). Stereoisomers of α-tocopheryl acetate. II. Biopotencies of all eight stereoisomers, individually or in mixtures, as determined by rat resorption-gestation tests. Int. J. Vitam. Nutr. Res..

[49] Hoppe P.P., Krennrich G. (2000). Bioavailability and potency of natural-source and all-racemic α-tocopherol in the human: A dispute. Eur. J. Nutr..

[50] Lodge J.K. (2005). Vitamin E bioavailability in humans. Proc. J. Plant Physiol..

[51] Weiser H., Vecchi M., Schlachter M. (1985). Stereoisomers of α-tocopheryl acetate. III. Simultaneous determination of resorption-gestation and myopathy in rats as a means of evaluating biopotency ratios of all-rac- and RRR-α-tocopheryl acetate. Int. J. Vitam. Nutr. Res..

[52] Jeanes Y.M., Hall W.L., Ellard S., Lee E., Lodge J.K. (2004). The absorption of vitamin E is influenced by the amount of fat in a meal and the food matrix. Br. J. Nutr..

[53] Bruno R.S., Leonard S.W., Park I.S., Zhao Y., Traber M.G. (2006). Human vitamin E requirements assessed with the use of apples fortified with deuterium-labeled α-tocopheryl acetate. Am. J. Clin. Nutr..

[54] Vinson J.A., Al Kharrat H., Andreoli L. (2005). Effect of Aloe vera preparations on the human bioavailability of vitamins C and E. Phytomedicine.

[55] Kemnic T.R., Coleman M. (2021). Vitamin E Deficiency-StatPearls-NCBI Bookshelf.

[56] Desmarchelier C., Tourniaire F., Nowicki M., Bott R., Borel P. (2015). How does vitamin E intake correlate with concentrations of tocopherols and their metabolites? Genetic variants involved in interindividual variability in vitamin E bioavailability. Free Radic. Biol. Med..

[57] Desmarchelier C., Tourniaire F., Nowicki M., Bott R., Borel P. (2015). The interindividual variability in vitamin E bioavailability in healthy male adults is significantly explained by a combination of SNPS in genes involved in vitamin E metabolism. Atherosclerosis.

[58] Dhakal S.P., He J. (2020). Microencapsulation of vitamins in food applications to prevent losses in processing and storage: A review. Food Res. Int..

[59] Julianto T., Yuen K.H., Noor A.M. (2000). Improved bioavailability of vitamin E with a self emulsifying formulation. Int. J. Pharm..

[60] Shishir M.R.I., Xie L., Sun C., Zheng X., Chen W. (2018). Advances in micro and nano-encapsulation of bioactive compounds using biopolymer and lipid-based transporters. Trends Food Sci. Technol..

[61] Ishwarya S.P., Anandharamakrishnan C., Stapley A.G.F. (2015). Spray-freeze-drying: A novel process for the drying of foods and bioproducts. Trends Food Sci. Technol..

[62] Parthasarathi S., Anandharamakrishnan C. (2016). Enhancement of oral bioavailability of vitamin E by spray-freeze drying of whey protein microcapsules. Food Bioprod. Process..

[63] Eid M., Sobhy R., Zhou P., Wei X., Wu D., Li B. (2020). β-cyclodextrin-soy soluble polysaccharide based core-shell bionanocomposites hydrogel for vitamin E swelling controlled delivery. Food Hydrocoll..

[64] Miyoshi N., Wakao Y., Tomono S., Tatemichi M., Yano T., Ohshima H. (2011). The enhancement of the oral bioavailability of γ-tocotrienol in mice by γ-cyclodextrin inclusion. J. Nutr. Biochem..

[65] Varga Z., Kosaras E., Komodi E., Katko M., Karpati I., Balla J., Paragh G., Aisa M.C., Galli F. (2008). Effects of tocopherols and 2,2’-carboxyethyl hydroxychromans on phorbol-ester-stimulated neutrophils. J. Nutr. Biochem..

[66] Jiang Z., Yin X., Jiang Q. (2011). Natural forms of vitamin E and 13’-carboxychromanol, a long-chain vitamin E metabolite, inhibit leukotriene generation from stimulated neutrophils by blocking calcium influx and suppressing 5-lipoxygenase activity, respectively. J. Immunol..

[67] Yam M.L., Abdul Hafid S.R., Cheng H.M., Nesaretnam K. (2009). Tocotrienols suppress proinflammatory markers and cyclooxygenase-2 expression in RAW264.7 macrophages. Lipids.

[68] Ng L.T., Ko H.J. (2012). Comparative effects of tocotrienol-rich fraction, alpha-tocopherol and alpha-tocopheryl acetate on inflammatory mediators and nuclear factor kappa B expression in mouse peritoneal macrophages. Food Chem..

[69] Qureshi A.A., Reis J.C., Papasian C.J., Morrison D.C., Qureshi N. (2010). Tocotrienols inhibit lipopolysaccharide-induced pro-inflammatory cytokines in macrophages of female mice. Lipids Health Dis..

[70] Jiang Q., Elson-Schwab I., Courtemanche C., Ames B.N. (2000). gamma-tocopherol and its major metabolite, in contrast to alpha-tocopherol, inhibit cyclooxygenase activity in macrophages and epithelial cells. Proc. Natl. Acad. Sci. USA.

[71] Wang Y., Jiang Q. (2013). gamma-Tocotrienol inhibits lipopolysaccharide-induced interlukin-6 and granulocyte colony-stimulating factor by suppressing C/EBPbeta and NF-kappaB in macrophages. J. Nutr. Biochem..

[72] Yang C., Jiang Q. (2019). Vitamin E delta-tocotrienol inhibits TNF-alpha-stimulated NF-kappaB activation by up-regulation of anti-inflammatory A20 via modulation of sphingolipid including elevation of intracellular dihydroceramides. J. Nutr. Biochem..

[73] Domazetovic V., Falsetti I., Viglianisi C., Vasa K., Aurilia C., Stio M., Menichetti S., Iantomasi T. (2021). Protective Role of Natural and Semi-Synthetic Tocopherols on TNFalpha-Induced ROS Production and ICAM-1 and Cl-2 Expression in HT29 Intestinal Epithelial Cells. Antioxidants.

[74] Elisia I., Kitts D.D. (2013). Modulation of NF-kappaB and Nrf2 control of inflammatory responses in FHs 74 Int. cell line is tocopherol isoform-specific. Am. J. Physiol. Gastrointest Liver Physiol..

[75] Ahn K.S., Sethi G., Krishnan K., Aggarwal B.B. (2007). Gamma-tocotrienol inhibits nuclear factor-kappaB signaling pathway through inhibition of receptor-interacting protein and TAK1 leading to suppression of antiapoptotic gene products and potentiation of apoptosis. J. Biol. Chem..

[76] Kannappan R., Yadav V.R., Aggarwal B.B. (2010). gamma-Tocotrienol but not gamma-tocopherol blocks STAT3 cell signaling pathway through induction of protein-tyrosine phosphatase SHP-1 and sensitizes tumor cells to chemotherapeutic agents. J. Biol. Chem..

[77] Wells S.R., Jennings M.H., Rome C., Hadjivassiliou V., Papas K.A., Alexander J.S. (2010). Alpha-, gamma- and delta-tocopherols reduce inflammatory angiogenesis in human microvascular endothelial cells. J. Nutr. Biochem..

[78] Wang Y., Moreland M., Wagner J.G., Ames B.N., Illek B., Peden D.B., Jiang Q. (2012). Vitamin E forms inhibit IL-13/STAT6-induced eotaxin-3 secretion by up-regulation of PAR4, an endogenous inhibitor of atypical PKC in human lung epithelial cells. J. Nutr. Biochem..

[79] Montagnani Marelli M., Marzagalli M., Moretti R.M., Beretta G., Casati L., Comitato R., Gravina G.L., Festuccia C., Limonta P. (2016). Vitamin E delta-tocotrienol triggers endoplasmic reticulum stress-mediated apoptosis in human melanoma cells. Sci. Rep..

[80] Xu M., Yang H., Zhang Q., Lu P., Feng Y., Geng X., Zhang L., Jia X. (2017). Alpha-Tocopherol prevents esophageal squamous cell carcinoma by modulating PPARgamma-Akt signaling pathway at the early stage of carcinogenesis. Oncotarget.

[81] Husain K., Francois R.A., Yamauchi T., Perez M., Sebti S.M., Malafa M.P. (2011). Vitamin E delta-tocotrienol augments the antitumor activity of gemcitabine and suppresses constitutive NF-kappaB activation in pancreatic cancer. Mol. Cancer Ther..

[82] Crispen P.L., Uzzo R.G., Golovine K., Makhov P., Pollack A., Horwitz E.M., Greenberg R.E., Kolenko V.M. (2007). Vitamin E succinate inhibits NF-kappaB and prevents the development of a metastatic phenotype in prostate cancer cells: Implications for chemoprevention. Prostate.

[83] Wang H., Hong J., Yang C.S. (2016). delta-Tocopherol inhibits receptor tyrosine kinase-induced AKT activation in prostate cancer cells. Mol. Carcinog..

[84] Galli F., Stabile A.M., Betti M., Conte C., Pistilli A., Rende M., Floridi A., Azzi A. (2004). The effect of alpha- and gamma-tocopherol and their carboxyethyl hydroxychroman metabolites on prostate cancer cell proliferation. Arch. Biochem. Biophys..

[85] Fontana F., Moretti R.M., Raimondi M., Marzagalli M., Beretta G., Procacci P., Sartori P., Montagnani Marelli M., Limonta P. (2019). delta-Tocotrienol induces apoptosis, involving endoplasmic reticulum stress and autophagy, and paraptosis in prostate cancer cells. Cell Prolif..

[86] Kaneko S., Sato C., Shiozawa N., Sato A., Sato H., Virgona N., Yano T. (2018). Suppressive Effect of Delta-Tocotrienol on Hypoxia Adaptation of Prostate Cancer Stem-like Cells. Anticancer Res..

[87] Elisia I., Kitts D.D. (2013). Different tocopherol isoforms vary in capacity to scavenge free radicals, prevent inflammatory response, and induce apoptosis in both adult- and fetal-derived intestinal epithelial cells. Biofactors.

[88] Elisia I., Kitts D.D. (2015). Tocopherol isoforms (alpha-, gamma-, and delta-) show distinct capacities to control Nrf-2 and NfkappaB signaling pathways that modulate inflammatory response in Caco-2 intestinal cells. Mol. Cell Biochem..

[89] Hidalgo M., Rodriguez V., Kreindl C., Porras O. (2020). Biological Redox Impact of Tocopherol Isomers Is Mediated by Fast Cytosolic Calcium Increases in Living Caco-2 Cells. Antioxidants.

[90] Campbell S.E., Stone W.L., Whaley S.G., Qui M., Krishnan K. (2003). Gamma (gamma) tocopherol upregulates peroxisome proliferator activated receptor (PPAR) gamma (gamma) expression in SW 480 human colon cancer cell lines. BMC Cancer.

[91] Idriss M., Hodroj M.H., Fakhoury R., Rizk S. (2020). Beta-Tocotrienol Exhibits More Cytotoxic Effects than Gamma-Tocotrienol on Breast Cancer Cells by Promoting Apoptosis via a P53-Independent PI3-Kinase Dependent Pathway. Biomolecules.

[92] Patacsil D., Tran A.T., Cho Y.S., Suy S., Saenz F., Malyukova I., Ressom H., Collins S.P., Clarke R., Kumar D. (2012). Gamma-tocotrienol induced apoptosis is associated with unfolded protein response in human breast cancer cells. J. Nutr. Biochem..

[93] Dronamraju V., Ibrahim B.A., Briski K.P., Sylvester P.W. (2019). gamma-Tocotrienol Suppression of the Warburg Effect Is Mediated by AMPK Activation in Human Breast Cancer Cells. Nutr. Cancer.

[94] Parajuli P., Tiwari R.V., Sylvester P.W. (2015). Anticancer Effects of gamma-Tocotrienol Are Associated with a Suppression in Aerobic Glycolysis. Biol. Pharm. Bull..

[95] Xu W., Mi Y., He P., He S., Niu L. (2017). gamma-Tocotrienol Inhibits Proliferation and Induces Apoptosis Via the Mitochondrial Pathway in Human Cervical Cancer HeLa Cells. Molecules.

[96] Peh H.Y., Tan W.S., Liao W., Wong W.S. (2016). Vitamin E therapy beyond cancer: Tocopherol versus tocotrienol. Pharmacol. Ther..

[97] Lee H., Lim Y. (2018). Tocotrienol-rich fraction supplementation reduces hyperglycemia-induced skeletal muscle damage through regulation of insulin signaling and oxidative stress in type 2 diabetic mice. J. Nutr. Biochem..

[98] Juretic N., Sepulveda R., D’Espessailles A., Vera D.B., Cadagan C., de Miguel M., Gonzalez-Manan D., Tapia G. (2021). Dietary alpha- and gamma-tocopherol (1:5 ratio) supplementation attenuates adipose tissue expansion, hepatic steatosis, and expression of inflammatory markers in a high-fat-diet-fed murine model. Nutrition.

[99] Allen L., Ramalingam L., Menikdiwela K., Scoggin S., Shen C.L., Tomison M.D., Kaur G., Dufour J.M., Chung E., Kalupahana N.S. (2017). Effects of delta-tocotrienol on obesity-related adipocyte hypertrophy, inflammation and hepatic steatosis in high-fat-fed mice. J. Nutr. Biochem..

[100] Pang K.L., Chin K.Y. (2019). The Role of Tocotrienol in Protecting Against Metabolic Diseases. Molecules.

[101] Kim Y., Natarajan S.K., Chung S. (2018). Gamma-Tocotrienol Attenuates the Hepatic Inflammation and Fibrosis by Suppressing Endoplasmic Reticulum Stress in Mice. Mol. Nutr. Food Res..

[102] Wong S.K., Chin K.Y., Suhaimi F.H., Ahmad F., Ima-Nirwana S. (2018). The effects of palm tocotrienol on metabolic syndrome and bone loss in male rats induced by high-carbohydrate high-fat diet. J. Funct. Foods.

[103] Zhao L., Kang I., Fang X., Wang W., Lee M.A., Hollins R.R., Marshall M.R., Chung S. (2015). Gamma-tocotrienol attenuates high-fat diet-induced obesity and insulin resistance by inhibiting adipose inflammation and M1 macrophage recruitment. Int. J. Obes..

[104] Shen C.L., Kaur G., Wanders D., Sharma S., Tomison M.D., Ramalingam L., Chung E., Moustaid-Moussa N., Mo H., Dufour J.M. (2018). Annatto-extracted tocotrienols improve glucose homeostasis and bone properties in high-fat diet-induced type 2 diabetic mice by decreasing the inflammatory response. Sci. Rep..

[105] Yuan J., Dong X., Yap J., Hu J. (2020). The MAPK and AMPK signalings: Interplay and implication in targeted cancer therapy. J. Hematol. Oncol..

[106] Li G., Lee M.J., Liu A.B., Yang Z., Lin Y., Shih W.J., Yang C.S. (2012). The antioxidant and anti-inflammatory activities of tocopherols are independent of Nrf2 in mice. Free Radic. Biol. Med..

[107] Wagner J.G., Birmingham N.P., Jackson-Humbles D., Jiang Q., Harkema J.R., Peden D.B. (2014). Supplementation with gamma-tocopherol attenuates endotoxin-induced airway neutrophil and mucous cell responses in rats. Free Radic. Biol. Med..

[108] McCary C.A., Abdala-Valencia H., Berdnikovs S., Cook-Mills J.M. (2011). Supplemental and highly elevated tocopherol doses differentially regulate allergic inflammation: Reversibility of alpha-tocopherol and gamma-tocopherol’s effects. J. Immunol..

[109] Lu G., Xiao H., Li G.X., Picinich S.C., Chen Y.K., Liu A., Lee M.J., Loy S., Yang C.S. (2010). A gamma-tocopherol-rich mixture of tocopherols inhibits chemically induced lung tumorigenesis in A/J mice and xenograft tumor growth. Carcinogenesis.

[110] Chen J.X., Liu A., Lee M.J., Wang H., Yu S., Chi E., Reuhl K., Suh N., Yang C.S. (2017). δ-and γ-tocopherols inhibit phIP/DSS-induced colon carcinogenesis by protection against early cellular and DNA damages. Mol. Carcinog..

[111] Prasad S., Gupta S.C., Tyagi A.K., Aggarwal B.B. (2016). gamma-Tocotrienol suppresses growth and sensitises human colorectal tumours to capecitabine in a nude mouse xenograft model by down-regulating multiple molecules. Br. J. Cancer.

[112] Hoffmann A., Natoli G., Ghosh G. (2006). Transcriptional regulation via the NF-kappaB signaling module. Oncogene.

[113] Shin J., Yang S.J., Lim Y. (2017). Gamma-tocopherol supplementation ameliorated hyper-inflammatory response during the early cutaneous wound healing in alloxan-induced diabetic mice. Exp. Biol. Med..

[114] Yamamoto Y., Enkhbaatar P., Sousse L.E., Sakurai H., Rehberg S.W., Asmussen S., Kraft E.R., Wright C.L., Bartha E., Cox R.A. (2012). Nebulization with gamma-tocopherol ameliorates acute lung injury after burn and smoke inhalation in the ovine model. Shock.

[115] Liu K.Y., Nakatsu C.H., Jones-Hall Y., Kozik A., Jiang Q. (2021). Vitamin E alpha- and gamma-tocopherol mitigate colitis, protect intestinal barrier function and modulate the gut microbiota in mice. Free Radic. Biol. Med..

[116] Lee H., Lim Y. (2019). Gamma-tocopherol ameliorates hyperglycemia-induced hepatic inflammation associated with NLRP3 inflammasome in alloxan-induced diabetic mice. Nutr. Res. Pract..

[117] Ozaki E., Campbell M., Doyle S.L. (2015). Targeting the NLRP3 inflammasome in chronic inflammatory diseases: Current perspectives. J. Inflamm. Res..

[118] Li X.H., Fu D., Latif N.H., Mullaney C.P., Ney P.H., Mog S.R., Whitnall M.H., Srinivasan V., Xiao M. (2010). Delta-tocotrienol protects mouse and human hematopoietic progenitors from gamma-irradiation through extracellular signal-regulated kinase/mammalian target of rapamycin signaling. Haematologica.

[119] Shibata A., Nakagawa K., Kawakami Y., Tsuzuki T., Miyazawa T. (2010). Suppression of gamma-tocotrienol on UVB induced inflammation in HaCaT keratinocytes and HR-1 hairless mice via inflammatory mediators multiple signaling. J. Agric. Food Chem..

[120] Johnson A.M., Kleczko E.K., Nemenoff R.A. (2020). Eicosanoids in Cancer: New Roles in Immunoregulation. Front. Pharmacol..

[121] Ju J., Hao X., Lee M.J., Lambert J.D., Lu G., Xiao H., Newmark H.L., Yang C.S. (2009). A gamma-tocopherol-rich mixture of tocopherols inhibits colon inflammation and carcinogenesis in azoxymethane and dextran sulfate sodium-treated mice. Cancer Prev. Res..

[122] Jiang Q., Jiang Z., Hall Y.J., Jang Y., Snyder P.W., Bain C., Huang J., Jannasch A., Cooper B., Wang Y. (2013). Gamma-tocopherol attenuates moderate but not severe colitis and suppresses moderate colitis-promoted colon tumorigenesis in mice. Free Radic. Biol. Med..

[123] Sanches L.D., Santos S.A., Carvalho J.R., Jeronimo G.D., Favaro W.J., Reis M.D., Felisbino S.L., Justulin L.A. (2013). Protective effect of gamma-tocopherol-enriched diet on N-methyl-N-nitrosourea-induced epithelial dysplasia in rat ventral prostate. Int. J. Exp. Pathol..

[124] Husain K., Centeno B.A., Chen D.T., Hingorani S.R., Sebti S.M., Malafa M.P. (2013). Vitamin E delta-tocotrienol prolongs survival in the LSL-KrasG12D/+;LSL-Trp53R172H/+;Pdx-1-Cre (KPC) transgenic mouse model of pancreatic cancer. Cancer Prev. Res..

[125] Husain K., Centeno B.A., Coppola D., Trevino J., Sebti S.M., Malafa M.P. (2017). delta-Tocotrienol, a natural form of vitamin E, inhibits pancreatic cancer stem-like cells and prevents pancreatic cancer metastasis. Oncotarget.

[126] Huang Y., Khor T.O., Shu L., Saw C.L., Wu T.Y., Suh N., Yang C.S., Kong A.N. (2012). A gamma-tocopherol-rich mixture of tocopherols maintains Nrf2 expression in prostate tumors of TRAMP mice via epigenetic inhibition of CpG methylation. J. Nutr..

[127] Wang H., Yang X., Liu A., Wang G., Bosland M.C., Yang C.S. (2018). delta-Tocopherol inhibits the development of prostate adenocarcinoma in prostate specific Pten-/- mice. Carcinogenesis.

[128] Huang P.H., Chuang H.C., Chou C.C., Wang H., Lee S.L., Yang H.C., Chiu H.C., Kapuriya N., Wang D., Kulp S.K. (2013). Vitamin E facilitates the inactivation of the kinase Akt by the phosphatase PHLPP1. Sci. Signal..

[129] Selvaduray K.R., Radhakrishnan A.K., Kutty M.K., Nesaretnam K. (2010). Palm tocotrienols inhibit proliferation of murine mammary cancer cells and induce expression of interleukin-24 mRNA. J. Interferon Cytokine Res..

[130] Siveen K.S., Ahn K.S., Ong T.H., Shanmugam M.K., Li F., Yap W.N., Kumar A.P., Fong C.W., Tergaonkar V., Hui K.M. (2014). Y-tocotrienol inhibits angiogenesis-dependent growth of human hepatocellular carcinoma through abrogation of AKT/mTOR pathway in an orthotopic mouse model. Oncotarget.

[131] Chang F., Steelman L.S., Shelton J.G., Lee J.T., Navolanic P.M., Blalock W.L., Franklin R., McCubrey J.A. (2003). Regulation of cell cycle progression and apoptosis by the Ras/Raf/MEK/ERK pathway (Review). Int. J. Oncol..

[132] Huang Y., Wu R., Su Z.Y., Guo Y., Zheng X., Yang C.S., Kong A.N. (2017). A naturally occurring mixture of tocotrienols inhibits the growth of human prostate tumor, associated with epigenetic modifications of cyclin-dependent kinase inhibitors p21 and p27. J. Nutr. Biochem..

[133] Blair C.A., Wu M., Huynh T., Hu H., Walia A., Yang C.S., Zi X. (2017). Delta tocopherol inhibits urothelial tumorigenesis in the UPII mutant Ha-ras transgenic mouse model and induces apoptosis via activation of the ATF4/CHOP-DR5 pathway. Proc. Am. Assoc. Cancer Res. Annu. Meet..

[134] Das Gupta S., So J.Y., Wall B., Wahler J., Smolarek A.K., Sae-Tan S., Soewono K.Y., Yu H., Lee M.J., Thomas P.E. (2015). Tocopherols inhibit oxidative and nitrosative stress in estrogen-induced early mammary hyperplasia in ACI rats. Mol. Carcinog..

[135] Wada S., Naito Y., Matsushita Y., Nouchi M., Kawai M., Minami E., Aoi W., Ikeda S., Higashi A., Yoshikawa T. (2017). δ-Tocotrienol suppresses tumorigenesis by inducing apoptosis and blocking the COX-2/PGE2 pathway that stimulates tumor–stromal interactions in colon cancer. J. Funct. Foods.

[136] Guan F., Li G., Liu A.B., Lee M.J., Yang Z., Chen Y.K., Lin Y., Shih W., Yang C.S. (2012). delta- and gamma-tocopherols, but not alpha-tocopherol, inhibit colon carcinogenesis in azoxymethane-treated F344 rats. Cancer Prev. Res..

[137] Li G.X., Lee M.J., Liu A.B., Yang Z., Lin Y., Shih W.J., Yang C.S. (2011). delta-tocopherol is more active than alpha-or gamma-tocopherol in inhibiting lung tumorigenesis in vivo. Cancer Prev. Res..

[138] Jiang Q. (2017). Natural Forms of Vitamin E as Effective Agents for Cancer Prevention and Therapy. Adv. Nutr..

[139] Jiang Q. (2014). Natural forms of vitamin E: Metabolism, antioxidant, and anti-inflammatory activities and their role in disease prevention and therapy. Free Radic. Biol. Med..

[140] Hernandez M.L., Wagner J.G., Kala A., Mills K., Wells H.B., Alexis N.E., Lay J.C., Jiang Q., Zhang H., Zhou H. (2013). Vitamin E, gamma-tocopherol, reduces airway neutrophil recruitment after inhaled endotoxin challenge in rats and in healthy volunteers. Free Radic. Biol. Med..

[141] Wiser J., Alexis N.E., Jiang Q., Wu W., Robinette C., Roubey R., Peden D.B. (2008). In vivo gamma-tocopherol supplementation decreases systemic oxidative stress and cytokine responses of human monocytes in normal and asthmatic subjects. Free Radic. Biol. Med..

[142] Vucinic L., Singh I., Spargo F.J., Hawley J.A., Linden M.D. (2010). Gamma tocopherol supplementation prevents exercise induced coagulation and platelet aggregation. Thromb Res..

[143] Liu M., Wallmon A., Olsson-Mortlock C., Wallin R., Saldeen T. (2003). Mixed tocopherols inhibit platelet aggregation in humans: Potential mechanisms. Am. J. Clin. Nutr..

[144] Mah E., Pei R., Guo Y., Ballard K.D., Barker T., Rogers V.E., Parker B.A., Taylor A.W., Traber M.G., Volek J.S. (2013). gamma-Tocopherol-rich supplementation additively improves vascular endothelial function during smoking cessation. Free Radic. Biol. Med..

[145] Lee I.M., Cook N.R., Gaziano J.M., Gordon D., Ridker P.M., Manson J.E., Hennekens C.H., Buring J.E. (2005). Vitamin E in the primary prevention of cardiovascular disease and cancer: The Women’s Health Study: A randomized controlled trial. JAMA.

[146] Pedrelli V.F., Lauriola M.M., Pigatto P.D. (2012). Clinical evaluation of photoprotective effect by a topical antioxidants combination (tocopherols and tocotrienols). J. Eur. Acad Dermatol. Venereol..

[147] Mahalingam D., Radhakrishnan A.K., Amom Z., Ibrahim N., Nesaretnam K. (2011). Effects of supplementation with tocotrienol-rich fraction on immune response to tetanus toxoid immunization in normal healthy volunteers. Eur. J. Clin. Nutr..

[148] Mah E., Noh S.K., Ballard K.D., Park H.J., Volek J.S., Bruno R.S. (2013). Supplementation of a gamma-tocopherol-rich mixture of tocopherols in healthy men protects against vascular endothelial dysfunction induced by postprandial hyperglycemia. J. Nutr. Biochem..

[149] Evans H.M., Bishop K.S. (1922). On the Existence of a Hitherto Unrecognized Dietary Factor Essential for Reproduction. Science.

[150] Barua S., Junaid M.A. (2015). Lifestyle, pregnancy and epigenetic effects. Epigenomics.

[151] Anderson K., Nisenblat V., Norman R. (2010). Lifestyle factors in people seeking infertility treatment—A review. Aust. N. Z. J. Obstet Gynaecol..

[152] Rumiris D., Purwosunu Y., Wibowo N., Farina A., Sekizawa A. (2006). Lower rate of preeclampsia after antioxidant supplementation in pregnant women with low antioxidant status. Hypertens Pregnancy.

[153] Traber M.G. (2014). Vitamin E inadequacy in humans: Causes and consequences. Adv. Nutr..

[154] Simsek M., Naziroglu M., Simsek H., Cay M., Aksakal M., Kumru S. (1998). Blood plasma levels of lipoperoxides, glutathione peroxidase, beta carotene, vitamin A and E in women with habitual abortion. Cell Biochem. Funct.

[155] Bastani P., Hamdi K., Abasalizadeh F., Navali N. (2011). Effects of vitamin E supplementation on some pregnancy health indices: A randomized clinical trial. Int. J. Gen. Med..

[156] Mohd Mutalip S.S., Ab-Rahim S., Rajikin M.H. (2018). Vitamin E as an Antioxidant in Female Reproductive Health. Antioxidants.

[157] Fang J.C., Kinlay S., Beltrame J., Hikiti H., Wainstein M., Behrendt D., Suh J., Frei B., Mudge G.H., Selwyn A.P. (2002). Effect of vitamins C and E on progression of transplant-associated arteriosclerosis: A randomised trial. Lancet.

[158] Salonen R.M., Nyyssonen K., Kaikkonen J., Porkkala-Sarataho E., Voutilainen S., Rissanen T.H., Tuomainen T.P., Valkonen V.P., Ristonmaa U., Lakka H.M. (2003). Six-year effect of combined vitamin C and E supplementation on atherosclerotic progression: The Antioxidant Supplementation in Atherosclerosis Prevention (ASAP) Study. Circulation.

[159] Gey K.F., Puska P., Jordan P., Moser U.K. (1991). Inverse correlation between plasma vitamin E and mortality from ischemic heart disease in cross-cultural epidemiology. Am. J. Clin. Nutr..

[160] Herrera E., Barbas C. (2001). Vitamin E: Action, metabolism and perspectives. J. Physiol. Biochem..

[161] Ashor A.W., Siervo M., Lara J., Oggioni C., Afshar S., Mathers J.C. (2015). Effect of vitamin C and vitamin E supplementation on endothelial function: A systematic review and meta-analysis of randomised controlled trials. Br. J. Nutr..

[162] May J.M. (2000). How does ascorbic acid prevent endothelial dysfunction?. Free Radic. Biol. Med..

[163] Ungvari Z., Kaley G., de Cabo R., Sonntag W.E., Csiszar A. (2010). Mechanisms of vascular aging: New perspectives. J. Gerontol. A Biol. Sci. Med. Sci..

[164] Pashkow F.J. (2011). Oxidative Stress and Inflammation in Heart Disease: Do Antioxidants Have a Role in Treatment and/or Prevention?. Int. J. Inflam..

[165] Traber M.G., Stevens J.F. (2011). Vitamins C and E: Beneficial effects from a mechanistic perspective. Free Radic. Biol. Med..

[166] Ward N.C., Wu J.H., Clarke M.W., Puddey I.B., Burke V., Croft K.D., Hodgson J.M. (2007). The effect of vitamin E on blood pressure in individuals with type 2 diabetes: A randomized, double-blind, placebo-controlled trial. J. Hypertens.

[167] Wu J.H., Ward N.C., Indrawan A.P., Almeida C.A., Hodgson J.M., Proudfoot J.M., Puddey I.B., Croft K.D. (2007). Effects of alpha-tocopherol and mixed tocopherol supplementation on markers of oxidative stress and inflammation in type 2 diabetes. Clin. Chem..

[168] Said E., Mousa S., Fawzi M., Sabry N.A., Farid S. (2021). Combined effect of high-dose vitamin A, vitamin E supplementation, and zinc on adult patients with diabetes: A randomized trial. J. Adv. Res..

[169] Liu Y., Ma C., Li P., Ma C., He S., Ping F., Zhang H., Li W., Xu L., Li Y. (2020). Potential Protective Effect of Dietary Intake of Non-alpha-Tocopherols on Cellular Aging Markers Mediated by Tumor Necrosis Factor-alpha in Prediabetes: A Cross-Sectional Study of Chinese Adults. Oxid. Med. Cell. Longev..

[170] Ebrahimi F.A., Samimi M., Foroozanfard F., Jamilian M., Akbari H., Rahmani E., Ahmadi S., Taghizadeh M., Memarzadeh M.R., Asemi Z. (2017). The Effects of Omega-3 Fatty Acids and Vitamin E Co-Supplementation on Indices of Insulin Resistance and Hormonal Parameters in Patients with Polycystic Ovary Syndrome: A Randomized, Double-Blind, Placebo-Controlled Trial. Exp. Clin. Endocrinol. Diabetes.

[171] Shirazi S.H., Pourghassem Gargari B., Izadi A., Taghizadeh S.H., Parizad M. (2021). Effect of Vitamin E on Serum Levels of Vascular Endothelial Growth Factor and Angiopoietin-1 in Women with Polycystic Ovary Syndrome: A Pilot Randomized, Placebo-Controlled Trial. Int. J. Fertil Steril..

[172] Carr B.R., Khan N., Adams-Huet B., Kakarla N., Havelock J.C., Gell J. (2006). Effect of vitamin E supplementation with and without hormone therapy on circulatory inflammatory markers in postmenopausal women. Fertil. Steril..

[173] Devaraj S., Li D., Jialal I. (1996). The effects of alpha tocopherol supplementation on monocyte function. Decreased lipid oxidation, interleukin 1 beta secretion, and monocyte adhesion to endothelium. J. Clin. Investig..

[174] Van Tits L.J., Demacker P.N., de Graaf J., Hak-Lemmers H.L., Stalenhoef A.F. (2000). alpha-tocopherol supplementation decreases production of superoxide and cytokines by leukocytes ex vivo in both normolipidemic and hypertriglyceridemic individuals. Am. J. Clin. Nutr..

[175] Stanger O., Herrmann W., Pietrzik K., Fowler B., Geisel J., Dierkes J., Weger M. (2003). DACH-LIGA homocystein (german, austrian and swiss homocysteine society): Consensus paper on the rational clinical use of homocysteine, folic acid and B-vitamins in cardiovascular and thrombotic diseases: Guidelines and recommendations. Clin. Chem. Lab. Med..

[176] Floegel A., Chung S.J., von Ruesten A., Yang M., Chung C.E., Song W.O., Koo S.I., Pischon T., Chun O.K. (2011). Antioxidant intake from diet and supplements and elevated serum C-reactive protein and plasma homocysteine concentrations in US adults: A cross-sectional study. Public Health Nutr..

[177] Saboori S., Shab-Bidar S., Speakman J.R., Yousefi Rad E., Djafarian K. (2015). Effect of vitamin E supplementation on serum C-reactive protein level: A meta-analysis of randomized controlled trials. Eur. J. Clin. Nutr..

[178] Somogyi A., Herold M., Kocsis I., Nagy G., Somfai G., Studinger P. (2005). Effect of vitamin E supplementation on the vitamin content of lipoprotein in young men and women. Orv. Hetil..

[179] Miller E.R., Pastor-Barriuso R., Dalal D., Riemersma R.A., Appel L.J., Guallar E. (2005). Meta-analysis: High-dosage vitamin E supplementation may increase all-cause mortality. Ann. Intern. Med..

[180] Chapman T.M., Kim H.J., Min D.B. (2009). Prooxidant activity of oxidized alpha-tocopherol in vegetable oils. J. Food Sci..

[181] Nadeem N., Woodside J.V., Kelly S., Allister R., Young I.S., McEneny J. (2012). The two faces of alpha- and gamma-tocopherols: An in vitro and ex vivo investigation into VLDL, LDL and HDL oxidation. J. Nutr. Biochem..

[182] Winterbone M.S., Sampson M.J., Saha S., Hughes J.C., Hughes D.A. (2007). Pro-oxidant effect of alpha-tocopherol in patients with type 2 diabetes after an oral glucose tolerance test--a randomised controlled trial. Cardiovasc. Diabetol..

[183] Himmelfarb J., Phinney S., Ikizler T.A., Kane J., McMonagle E., Miller G. (2007). Gamma-tocopherol and docosahexaenoic acid decrease inflammation in dialysis patients. J. Ren. Nutr..

[184] Tasanarong A., Vohakiat A., Hutayanon P., Piyayotai D. (2013). New strategy of alpha- and gamma-tocopherol to prevent contrast-induced acute kidney injury in chronic kidney disease patients undergoing elective coronary procedures. Nephrol. Dial. Transplant..

[185] Koay Y.Y., Tan G.C.J., Phang S.C.W., Ho J.I., Chuar P.F., Ho L.S., Ahmad B., Abdul Kadir K. (2021). A Phase IIb Randomized Controlled Trial Investigating the Effects of Tocotrienol-Rich Vitamin E on Diabetic Kidney Disease. Nutrients.

[186] Ascherio A., Weisskopf M.G., O’Reilly E.J., Jacobs E.J., McCullough M.L., Calle E.E., Cudkowicz M., Thun M.J. (2005). Vitamin E intake and risk of amyotrophic lateral sclerosis. Ann. Neurol..

[187] Adalier N., Parker H. (2016). Vitamin E, Turmeric and Saffron in Treatment of Alzheimer’s Disease. Antioxidants.

[188] Morris M.C., Evans D.A., Tangney C.C., Bienias J.L., Wilson R.S., Aggarwal N.T., Scherr P.A. (2005). Relation of the tocopherol forms to incident Alzheimer disease and to cognitive change. Am. J. Clin. Nutr..

[189] Morris M.C., Schneider J.A., Li H., Tangney C.C., Nag S., Bennett D.A., Honer W.G., Barnes L.L. (2015). Brain tocopherols related to Alzheimer’s disease neuropathology in humans. Alzheimers Dement..

[190] Baldeiras I., Santana I., Proenca M.T., Garrucho M.H., Pascoal R., Rodrigues A., Duro D., Oliveira C.R. (2008). Peripheral oxidative damage in mild cognitive impairment and mild Alzheimer’s disease. J. Alzheimers Dis..

[191] Georgousopoulou E.N., Panagiotakos D.B., Mellor D.D., Naumovski N. (2017). Tocotrienols, health and ageing: A systematic review. Maturitas.

[192] Mangialasche F., Xu W., Kivipelto M., Costanzi E., Ercolani S., Pigliautile M., Cecchetti R., Baglioni M., Simmons A., Soininen H. (2012). Tocopherols and tocotrienols plasma levels are associated with cognitive impairment. Neurobiol. Aging..

[193] Mangialasche F., Solomon A., Kareholt I., Hooshmand B., Cecchetti R., Fratiglioni L., Soininen H., Laatikainen T., Mecocci P., Kivipelto M. (2013). Serum levels of vitamin E forms and risk of cognitive impairment in a Finnish cohort of older adults. Exp. Gerontol.

[194] Dysken M.W., Sano M., Asthana S., Vertrees J.E., Pallaki M., Llorente M., Love S., Schellenberg G.D., McCarten J.R., Malphurs J. (2014). Effect of vitamin E and memantine on functional decline in Alzheimer disease: The TEAM-AD VA cooperative randomized trial. JAMA.

[195] Pavlik V.N., Doody R.S., Rountree S.D., Darby E.J. (2009). Vitamin E use is associated with improved survival in an Alzheimer’s disease cohort. Dement. Geriatr Cogn Disord..

[196] Arlt S., Muller-Thomsen T., Beisiegel U., Kontush A. (2012). Effect of one-year vitamin C- and E-supplementation on cerebrospinal fluid oxidation parameters and clinical course in Alzheimer’s disease. Neurochem. Res..

[197] Devore E.E., Grodstein F., van Rooij F.J., Hofman A., Stampfer M.J., Witteman J.C., Breteler M.M. (2010). Dietary antioxidants and long-term risk of dementia. Arch. Neurol..

[198] Zandi P.P., Anthony J.C., Khachaturian A.S., Stone S.V., Gustafson D., Tschanz J.T., Norton M.C., Welsh-Bohmer K.A., Breitner J.C., Cache County Study G. (2004). Reduced risk of Alzheimer disease in users of antioxidant vitamin supplements: The Cache County Study. Arch. Neurol..

[199] Giraldo E., Lloret A., Fuchsberger T., Vina J. (2014). Abeta and tau toxicities in Alzheimer’s are linked via oxidative stress-induced p38 activation: Protective role of vitamin E. Redox Biol..

[200] Petersen R.C., Thomas R.G., Grundman M., Bennett D., Doody R., Ferris S., Galasko D., Jin S., Kaye J., Levey A. (2005). Vitamin E and donepezil for the treatment of mild cognitive impairment. N. Engl. J. Med..

[201] Kang J.H., Cook N., Manson J., Buring J.E., Grodstein F. (2006). A randomized trial of vitamin E supplementation and cognitive function in women. Arch. Intern. Med..

[202] Sano M., Ernesto C., Thomas R.G., Klauber M.R., Schafer K., Grundman M., Woodbury P., Growdon J., Cotman C.W., Pfeiffer E. (1997). A controlled trial of selegiline, alpha-tocopherol, or both as treatment for Alzheimer’s disease. The Alzheimer’s Disease Cooperative Study. N. Engl. J. Med..

[203] Abner E.L., Schmitt F.A., Mendiondo M.S., Marcum J.L., Kryscio R.J. (2011). Vitamin E and all-cause mortality: A meta-analysis. Curr. Aging Sci..

[204] Chin S.F., Ibahim J., Makpol S., Abdul Hamid N.A., Abdul Latiff A., Zakaria Z., Mazlan M., Mohd Yusof Y.A., Abdul Karim A., Wan Ngah W.Z. (2011). Tocotrienol rich fraction supplementation improved lipid profile and oxidative status in healthy older adults: A randomized controlled study. Nutr. Metab..

[205] Rondanelli M., Faliva M.A., Peroni G., Moncaglieri F., Infantino V., Naso M., Perna S. (2015). Focus on Pivotal Role of Dietary Intake (Diet and Supplement) and Blood Levels of Tocopherols and Tocotrienols in Obtaining Successful Aging. Int. J. Mol. Sci..

[206] Michaelsson K., Wolk A., Byberg L., Arnlov J., Melhus H. (2014). Intake and serum concentrations of alpha-tocopherol in relation to fractures in elderly women and men: 2 cohort studies. Am. J. Clin. Nutr..

[207] Holvik K., Gjesdal C.G., Tell G.S., Grimnes G., Schei B., Apalset E.M., Samuelsen S.O., Blomhoff R., Michaelsson K., Meyer H.E. (2014). Low serum concentrations of alpha-tocopherol are associated with increased risk of hip fracture. A NOREPOS study. Osteoporos Int..

[208] D’Adamo C.R., Shardell M.D., Hicks G.E., Orwig D.L., Hochberg M.C., Semba R.D., Yu-Yahiro J.A., Ferrucci L., Magaziner J.S., Miller R.R. (2011). Serum vitamin E concentrations among highly functioning hip fracture patients are higher than in nonfracture controls. Nutr. Res..

[209] Pette D., Spamer C. (1986). Metabolic properties of muscle fibers. Fed Proc..

[210] Evans W.J. (2000). Vitamin E, vitamin C, and exercise. Am. J. Clin. Nutr..

[211] Ju J., Picinich S.C., Yang Z., Zhao Y., Suh N., Kong A.N., Yang C.S. (2010). Cancer-preventive activities of tocopherols and tocotrienols. Carcinogenesis.

[212] Klein E.A., Thompson I.M., Tangen C.M., Crowley J.J., Lucia M.S., Goodman P.J., Minasian L.M., Ford L.G., Parnes H.L., Gaziano J.M. (2011). Vitamin E and the risk of prostate cancer: The Selenium and Vitamin E Cancer Prevention Trial (SELECT). JAMA.

[213] Nesaretnam K., Selvaduray K.R., Abdul Razak G., Veerasenan S.D., Gomez P.A. (2010). Effectiveness of tocotrienol-rich fraction combined with tamoxifen in the management of women with early breast cancer: A pilot clinical trial. Breast Cancer Res..

